# Baseline proteomics characterisation of the emerging host biomanufacturing organism *Halomonas bluephagenesis*

**DOI:** 10.1038/s41597-022-01610-0

**Published:** 2022-08-13

**Authors:** Matthew Russell, Andrew Currin, William Rowe, Guo-Qiang Chen, Perdita Barran, Nigel S. Scrutton

**Affiliations:** 1 Manchester Institute of Biotechnology and Dept. of Chemistry, The University of Manchester, Manchester, M1 7DN UK; 2 School of Life Sciences, Tsinghua University, 100084 Beijing, China

**Keywords:** Proteomics, Metabolic engineering

## Abstract

Despite its greener credentials, biomanufacturing remains financially uncompetitive compared with the higher carbon emitting, hydrocarbon-based chemical industry. Replacing traditional chassis such as *E. coli* with novel robust organisms, are a route to cost reduction for biomanufacturing. Extremophile bacteria such as the halophilic *Halomonas bluephagenesis* TD01 exemplify this potential by thriving in environments inherently inimical to other organisms, so reducing sterilisation costs. Novel chassis are inevitably less well annotated than established organisms. Rapid characterisation along with community data sharing will facilitate adoption of such organisms for biomanufacturing. The data record comprises a newly sequenced genome for the organism and evidence via LC-MS based proteomics for expression of 1160 proteins (30% of the proteome) including baseline quantification of 1063 proteins (27% of the proteome), and a spectral library enabling re-use for targeted LC-MS proteomics assays. Protein data are annotated with KEGG Orthology, enabling rapid matching of quantitative data to pathways of interest to biomanufacturing.

## Abstract (summary)

**Table:** 

Measurement(s)	Genome • Proteomic Profile
Technology Type(s)	PacBio Sequel System • nanoflow liquid chromatography-electrospray ionisation mass spectrometry
Sample Characteristic - Organism	Halomonas sp. TD01

## Background & Summary

Biomanufacturing remains financially uncompetitive compared with the production of chemical products from hydrocarbon feed stocks. Development of cost-competitive biomanufacturing routes for chemicals will add to efforts to achieve a carbon neutral economy. Displacement of fossil fuels from the energy market, currently underway, may dramatically change the economics around hydrocarbon extraction leading to price fluctuation in many of the commodity chemicals derived from them. Alternative sources of commodity chemicals at prices comparable to those currently offered by hydrocarbon based industry is therefore critical to sustain industries dependent on these chemicals in coming decades^1^.

Alternative organisms to those traditionally employed in biomanufacturing (e.g., *Escherichia coli*, *Saccharomyces cerevisiae*) either found in nature, or developed in the laboratory, may have features that enable major reductions in production costs. One such organism is *Halomonas bluephagenesis*^2–8^. This is a marine and salt lake inhabiting *Halomonas* genus of bacteria, which is optimally cultured in high salt and high pH media, thereby removing the need for costly sterilisation of equipment^2^. The suitability of *Halomonas* for biomanufacturing has been demonstrated, for example in production of polyhydroxyalkanoates^6,9^, and L-Threonine^10^ as well as its native bioplastic ectoine^7^ however it is yet to achieve commercial exploitation. There is a need to rapidly characterise and gain engineered control over such organisms if they are to transition from discovery science to industrial application.

Systems biology tools will play a vital role in achieving the required engineered control^11^ and support the iterative Design, Build, Test, Learn processes to achieve desired production^12^. Genomes may be readily sequenced and proteomes catalogued and shared. Proteins provide the machinery which sustain the organisms and enable it to produce required compounds. Understanding how protein expression shifts during industrial scale processing will support the adaptation of manufacturing facilities to the novel organisms. Understanding potential interactions between inserted and native proteins including competitive side reactions of both precursor and product molecules will support attainment of desirable outcomes in biomanufacturing projects. Shared proteomics resources will provide the biomanufacturing community with ready access to such modern systems biology tools and support innovation and accelerate industrial application of these organisms to next-generation industrial biotechnology^13^.

**Fig. 1:**
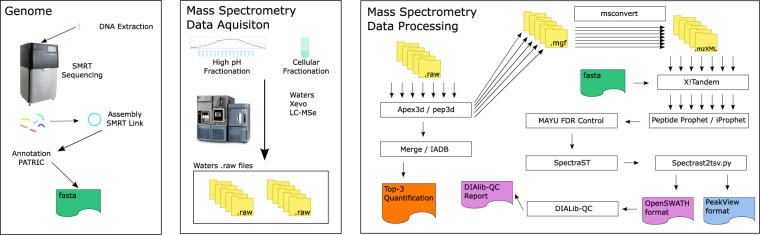
Schematic of data acquisition and processing pipeline. Left: Acquisition of genome; Middle: Acquisition of mass spectrometry data; Right: Data processing pipeline.

The acquired *H. bluephagenesis* genome sequence data has been deposited^14^ at the European Nucleotide Archive (ENA)^15^. The deposition includes all sequence reads and the assembled a functionally annotated circularised genome. A proteomics resource comprising a catalogue of protein expression in the late-log/early stationary growth phase and associated spectral library suitable for re-use in targeted proteomics experiments is deposited at PRIDE^16^. Quality of the proteomics dataset is assured by controlling against a parallel acquisition of data for the model organism and the established biomanufacturing chassis *E. coli*. The control data set is compared against available public resources to enable estimation of the quality of data for *H. bluephagenesis*. The expression of a third of annotated genes is confirmed at the protein level. Cellular protein abundance as estimated by MS^e ^^17^ Top3^18,19^ method are tabulated for both *E. coli* and *H. bluephagenesis* as molar abundance per unit dry mass. A spectral library for all peptides identified during the analysis is supplied to facilitate targeted proteomics analysis. All the raw data, processing scripts, and processed spectral libraries are available on the EBI Pride^20,21^ repository^16^. Spectral libraries have been prepared in each of a set of widely used formats to facilitate reuse with the most widely used processing pipelines: .sptxt and .splib for spectrast and skyline; .peakview.tsv for Sciex software; and .openms.tsv for openms and openSWATH. The *Halomonas* protein fasta database used is provided for reference. For a schematic of the data acquisition and processing pipeline see Fig. 1.

## Methods

### Bacteria strains

The *Halomonas bluephagenesis* TD01 strain investigated here was isolated from Aydingkol Lake in Xinjiang, China in 2009^2^ and has been under continuing development for biomanufacturing since its isolation^2–8,22^. *E. coli* (K12/MG1655) was processed in identical manner for each process and used as a control for comparison against public datasets.

### Genome sequencing and proteome library generation

Genomic DNA for *H. bluephagenesis* TD01 strain was extracted and purified using the Monarch genomic DNA purification kit (New England Biolabs) following the manufacturer’s instructions. The gDNA was then sheared using a gTube (Covaris) and prepared for sequencing following the SMRTbell Express Template Preparation Kit 2.0 protocol. Sequencing data were obtained using the Sequel system (Pacific Biosciences) with a 10-hour acquisition. *De novo* assembly of the TD01 genome was performed using the Microbial assembly algorithm in SMRT Link, which generated a single circular contig assembly, representing the complete TD01 genome. The assembly was analysed and annotated with the PATRIC online tool^23^.

### Media

Luria broth as standard for *E. coli* and adjusted to 5% sodium chloride, pH 9 for *H. bluephagenesis* was prepared as follows. LB Broth Miller was prepared from powder (Formedium, 25 g L^−1^ comprising tryptone 10 g L^−1^, yeast extract 5 g L^−1^ and sodium chloride 10 g L^−1^, pH 7.0). For *H. bluephagenesis* this was adjusted to 5% sodium chloride (additional 40 g L^−1^) and pH adjusted with sodium hydroxide (5 M, 4 mL L^−1^, to pH 9). Liquid media was sterilised by autoclave. For plates, agar (15 g L^−1^) was added to the appropriate sterile low- or high-salt media described above and heated in a microwave (30% power, 20 min) then poured into plates and allowed to cool in a laminar flow hood. Plates were stored (5 °C) for up to four weeks prior to use.

### Plate cell cultures

Cells from a glycerol stock were aseptically inoculated onto agar media plates prepared as above. Inoculated plates were incubated (37 °C, overnight) then transferred to cold storage (4 °C). Colonies were taken to inoculate liquid cell cultures, see below, within four days.

### Liquid cell cultures

A single bacterial colony of either *E. coli* K12 or *H. bluephagenesis* TD01 was transferred aseptically to media (50 mL) of the required type (see above) in a 250 mL conical flask. Cultures were incubated (37 °C, 200 rpm) and OD_600_ monitored.

### Pellet collection for whole cell analysis

When OD_600_ was 0.3–0.6, which prior experiments indicated growth to be in the late log/early stationary phase, the culture (450 µL) was recovered by placing into pre-weighed 5 mL microfuge tubes on ice and pelleted by centrifugation (3200 × g, 10 min, 4 °C). The supernatant was discarded. Wet mass pellet was determined by difference with tube empty mass. Pellets were frozen (−20 °C) for later whole cell digestion.

### Pellet wet to dry mass estimation

A set of six pellets each of *E. coli* and *H. bluephagenesis* TD01 were collected as above. Pellets were heated (70 °C, overnight) to evaporate water. The dry mass of the pellet was then determined. Pellets were subject to a further six hours of drying and re-weighting to insure dry mass had reached an end point. The relationship between wet and dry mass was established by linear regression.

### Cell pellet lysis

Frozen pellets were simultaneously resuspended and defrosted in S-trap lysis buffer (7% SDS, 70 mM TEAB, pH 7.55, 3 µL buffer per mg wet mass of pellet). DNA was removed by addition of bensonase (Sigma/Merk, 1 µL) and sample vortexed briefly. If required, a second addition of bensonase removed any remaining viscosity. Cell debris were removed by centrifugation (4200 × g, 2 min).

### Cell fractionation to periplasm and plasma membrane

Bacteria were cultured as above. At OD_600_ 0.3–0.6 an OD10 sample (vol in mL = 10/ OD_600_) was recovered to a 5 mL microfuge tube and pelleted (3200 × g, 10 min, 4 °C). Working on ice pellet was resuspended in ice cold “buffer 1” (500 µL, 500 mM sucrose, 5 mM EDTA, for *Halomonas* only – 5% sodium chloride, 100 mM Tris-acetate pH 8.2). Lysozyme (40 µL, 2 mg mL^−1^) was added to the suspension, quickly followed by dilution with further ice-cold water (500 µL) then incubated (5 min, on ice). Magnesium sulfate (20 µL, 1 M) was added stripping the outer membrane and periplasm from the bacteria leaving intact spheroplasts. The spheroplasts were pelleted by ultracentrifugation (14,000 rpm, 10 min, 4 °C); both pellet (spheroblast) and supernatant (periplasm) were retained. The supernatant was transferred to a fresh microfuge tube and remaining debris pelleted by centrifugation (14,000 rpm, 10 min, 4 °C), the top fraction of the resulting supernatant (500 µL) was combined with 2 × S-trap SDS-lysis buffer (500 µL, 10% SDS, 100 mM TEAB pH 7.55). Spheroplasts were washed twice by gentle resuspension in ice-cold “buffer 2” (1 mL, 250 mM sucrose, 120 mM MgSO_4_, 50 mM Tris-acetate pH 8.2). Spheroplasts were resuspended in ice-cold “buffer 3” (1 mL, 2.5 mM EDTA, 50 mM Tris-acetate pH 8.2) supplemented with bensoase (Sigma, 1 µL, 250 units/µL). Spheroplasts were disrupted by sonication on ice (3 × 15 s burst, 25% power, 3 mm diameter tipped probe). The membranes and cytoplasm were separated by ultracentrifugation (202,000 × g, 30 min, 4 °C) following which the supernatant containing the cytoplasm was discarded, the membrane resuspended in “buffer 3” and ultracentrifugation repeated to produce a clean membrane fraction which was resuspended in 1 × S-trap lysis buffer (2 mL, 5% SDS, 500 mM TEAB pH 7.55).

### Protein digest

Total protein was quantified by micro-BCA assay (Pierce) according to the manufacturer’s instructions. Protein was digested with the s-trap mini kit (Protifi) according to the manufacturer’s instructions, the sample already being in the recommended SDS-lysis buffer (see above). Briefly, sample volume was taken to give 1–5 mg total protein, reduced with TCEP (50 mM final concentration) and incubated (60 °C, 30 min), alkylated with iodoacetamide (40 mM final concentration) and incubated at room temperature in the dark for 30 min. The sample was acidified with phosphoric acid (1/10 vol, 12%) and diluted in s-trap binding buffer (7 vols, 90% methanol, 100 mM TEAB). The sample was loaded onto s-trap in cycles of 190 µL aliquots followed by centrifugation (4,000 × g, 1 min). The s-trap was washed (4 × 150 µL) with centrifugation as for loading. Protein was digested by addition of trypsin (Promega, 20 µL, 0.5 µg µL^−1^) in digestion buffer (50 mM TEAB) and incubated (47 °C, 1 hr) in a water bath.

### Offline high pH fractionation

Whole cell lysate digests were fractionated by offline high-pH fractionation using an Agilent 1200 series LC system with a C-18 reverse phase column (Agilent ZORBAX 300Extend-C18 4.6 × 150 mm, 3.5 µm particle size) in a column oven (45 °C) with mobile phases A (Water, 0.1% ammonium hydroxide) and B (acetonitrile, 0.1% ammonium hydroxide). The system was equilibrated (1 mL min^−1^, 3% B), the sample injected and system held at starting conditions for 75 min or until the UV trace at 214 nm had returned to baseline. Peptides were eluted with a linear gradient (25 min, 0.75 mL min^−1^, 3–27% B), followed by washing the column (5 min, 0.75 mL min^−1^, 50% B) then a further wash (5 min, 0.75 mL min^−1^, 100% B) followed by re-equilibration with starting conditions. Peptides fractions were collected (9–40 min run time, 0.35 min per fraction) into a 96 well plate. The plate was lyophilised, samples were resuspended in LC-MS loading buffer (3% acetonitrile, 0.1% formic acid, iRT peptides) and fractions pooled in threes taken from begging, middle and end of the run. Pooled samples were processed by LC-MS (see below).

### Biognosis iRT for retention time calibration and quantification

Retention time index peptides^24^ (JPT) were spiked into samples prior to LC-MS to enable both retention time normalisation and TOP3 quantification (see Table 1). The peptides comprise a common community standard set used for such normalisation. Combined volumes of sample, iRT stock and injection volumes were adjusted so that the amount injected into the LC-MS column was held constant between samples. Of the 11 iRT peptides, five were injected at approximately 434 fmol on column and used for TOP3 quantification, as indicated in the table.

**Table 1:** Table of iRT standard peptides.

Sequence	Stock concentration/pmol µL^−1^	Amount on column/pmol	Used for Quantification
LFLQFGAQGSPFLK	1.50	0.15	
YILAGVENSK	1.62	0.16	X
TPVISGGPYEYR	1.63	0.16	X
LGGNEQVTR	1.67	0.17	X
VEATFGVDESNAK	1.67	0.17	X
GAGSSEPVTGLDAK	1.68	0.17	X
TPVITGAPYEYR	2.90	0.29	
GTFIIDPGGVIR	3.20	0.32	
DGLDAASYYAPVR	3.89	0.39	
ADVTPADFSEWSK	5.00	0.5	
GTFIIDPAAVIR	15.00	1.5	

### LC-MS

Mass spectrometry data were acquired on a Waters Xevo G2S QToF coupled to a nanoAquity liquid chromatography system in trap-elute mode. The sample was injected onto a trap column (Waters nanoEase, 100 Å, 5 µm, 180 µm × 20 mm) with 3% acetonitrile, 0.1% formic acid at 0.6 mL min^−1^ then eluted through an analytical column (Waters nanoEase M/Z Peptide CSH C18, 130 Å, 1.7 µm, 75 µm × 150 mm) with an 80 minute linear gradient of 3–45% acetonitrile, 0.1% formic acid at 0.6 µL min^−1^ followed by column wash and re-equilibration for a total injection run time of 90 min. Data were acquired in MS^e^ mode with leucine enkephalin lockspray mass correction at 60 s intervals, over m/z 50–2000 with 1 s scan times, low energy scans were acquired with 5 V collision energy and high energy scans with a ramp of 20–35 V collision energy.

### Data processing

The workflow for spectral library generation was scripted using gnu-make. Both the script and detailed accompanying documentation are included in the Pride submission and available on git-hub (https://github.com/M-Russell/HalomonasProcessingRepository). The documentation describes in detail the proteomics workflow managed by the gnu-make script including every command line used to process raw data to final outputs. In brief, proprietary Waters processing tools Apex3d and peptdie3d were used to process Waters raw MS^e^ data into mass spectra. These spectra were then processed through two separate pathways. One route merged the spectral data for each species *H. bluephagenesis* and *E. coli* then searched and quantified proteins using Waters IADB search program with TOP3 quantification. The other route took spectral data in mgf format exported from the peptide3d program and processed the spectra through the Trans-Proteomic-Pipeline^25^ matching spectra to peptides with X!Tandem^26^, controlling for false discovery rates using Peptide Prophet, iProphet and Mayu^27^ and producing a spectral library with spectrast in a readily accessible format directly compatible with the widely used Skyline^28^ software. The spectral library was subsequently converted into tab delimited version compatible with Openswath^29^ and Sciex peakview software. The quality of the spectral library was assessed with the DIAlib-QC tool. Proteins quantified via TOP3 quantification with IADB were filtered for those identified at less than 1% FDR by Maye.

### KEGG orthology annotation

The protein fasta database was broken into four parts and each part was uploaded to KEGG BlastKOALA^30^ KO assignment tool (https://www.kegg.jp/kegg/mapper/assign_ko.html) with the family/genus to be searched set to *Halomonas* (2745). The resulting tables of matched protein annotation KO number were concatenated together.

### KEGG pathway reconstruction

Kegg KO numbers assigned to proteins above were filtered for those assigned to quantified proteins. This list was submitted to the Kegg Reconstruction Tool^31^ (https://www.genome.jp/kegg/mapper). The resulting “Pathway” and “Module” tables were copied to excel files and are included as supplementary information.

### Tabulated summary of baseline expression

A tabulated summary of the data acquired above was prepared for *H. bluephagenesis* as follows. The sequence annotations from each of the protein sequences in the fasta file were put in the left hand column. This tabulation was followed by columns indicating first if peptides from the protein are present in the spectral library, which also indicates evidence of protein expression at the protein level, and second if the protein was quantified. Where protein sequences were matched to Kegg entries via BlastKOALA as described above details of the KO number and description were entered in the next two columns. The final column contained the estimate of protein expression in mmol per gram dry mass of bacterial pellet. This estimate was obtained by adjusting the TOP3 estimate of protein amount in fmol on column for the fraction of total protein taken for the digest, the fraction of the digest taken for offline fractionation and the fraction of fractionated material injected on the LC-MS system.

## Data Records

The data submissions comprise both genome sequence and proteomics data.

The genome sequence data is available through the European Nucleotide Archive (ENA)^14^ in a deposition that included all sequence reads obtained in the project and the processed and functionally annotated genome.

A set of proteomics resources^16^ is deposited in the Pride repository for *Halomonas bluephagenesis* TD01 and equivalent quality control data set derived in the same manner from *E. coli* K12. The deposition includes all the raw mass spectrometry data and key intermediate data types. The raw cell sample data In Waters .raw format. Processed spectra are included as .mzXML, search results are included as both .pep.xml and .mzid format. Spectral libraries for peptides identified is included in .sptxt, .traml, and both openms and peakview.tsv covering formats used by the majority of DIA proteomics analysis software to facilitate re-use.

In addition to raw proteomics data the deposition includes a summary table for each species (ecoli_proteomeQuantTable.csv and halo_proteomeQuantTable.csv). These table note against each protein or gene identified in the protein sequence database for the analysis whether that entry is included in the spectral library, or has been quantified, where it has been matched against the kegg database its Kegg orthology and description is given, where it has been quantified its estimated abundance in mmol gram^−1^ by dry mass is given.

Finally the deposit includes the documented gnu-make script also available on authors github page (https://github.com/M-Russell/HalomonasProcessingRepository), to ensure reproducibility of the data processing pipeline.

## Technical Validation

### Confirmed need for *H. bluephagenesis* spectral library

The *Halomonas* genus of bacteria, of which *H. bluephagenesis* is an example are members of the salt tolerant Oceanospirillales order of the Proteobacteria phylum. Although the phylum Proteobacteria includes the model bacterium *E. coli* the two species are quite distantly related and adapted to quite different environments: *Halomonas* to salt waters of seas, lakes and estuaries and *Escherichia* to the lower intestines of warm-blooded organisms. A comparison of the theoretical tryptic digests of the complete *H. bluephagenesis* and *E. coli* protein databases used here show only 12% of *H. bluephagenesis* peptides are found in *E. coli*. Of the 7816 distinct modified peptide sequences in the *H. bluephagenesis* library only 111 appear in the Mortiz *E. coli* library^32^. Both these observations confirm the need for species specific proteomic resources for these bacteria.

### Genome and annotation

The *H. bluephagenesis* genome was assembled *de novo* using the SMRT Link Microbial Assembly algorithm from roughly 2 gigabases of sequencing data. The assembly produced three contigs (4,142,577, 3,071 and 1,925), with the largest identified as circular and which represents the TD01 genome. The assembly had an average of 400-fold coverage across each contig.

### Data quality control

The proteomics resources presented here for *Halomonas bluephagenesis* TD01 comprises a catalogue of proteins with evidence of expression at a protein level; a subset of those proteins with expression estimated in terms of copy numbers per cell; and a spectral library for identified peptides to enable re-use of data in data independent and targeted quantitative proteomics experiments. The quality of these resources has been verified by acquiring equivalent data for model bacteria *E. coli* K12 alongside *H. bluephagenesis* TD01. Comparison of the *E. coli* data with pubic resources such a recently published spectral library^32^ (termed the Moritz library below) and the paxDB^33^ (https://pax-db.org) comprehensive absolute protein abundance database provides assurance of the quality of the data acquisition and processing pipeline.

### Control of false positive rate

The principle quality criteria for the expressed protein catalogue, quantified protein catalogue and spectral library is the false discovery rate (FDR). False protein discoveries in the catalogues represent false data points. More significantly for re-use of the spectral library data false peptide-spectrum matches (PSM) with false iRT annotations threaten future analysis based on the library.

**Fig. 2:**
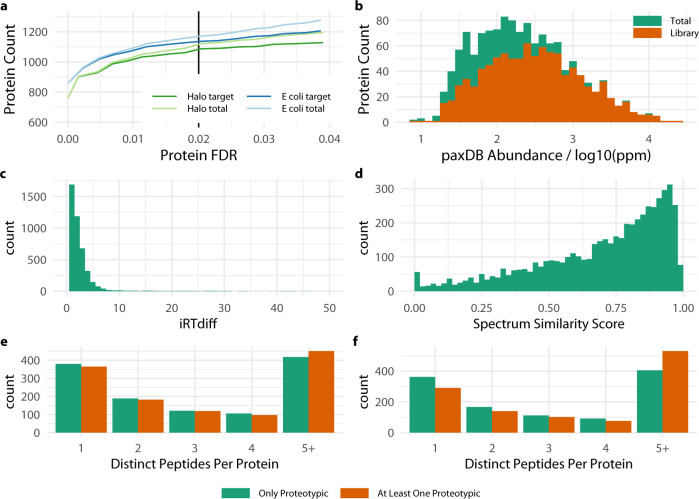
Quality metrics for protein identification and spectral library. (**a**) Plot of target and total (target and decoy) protein identifications at given false discovery rate (FDR), 2% cut-off is indicated. (**b**) Depth of coverage of *E. coli* quantified in the paxDB database. (**c**) Distribution of disparity in iRT index for to *E. coli* peptides in this analysis with that published by Moritz *et al*. (**d**) Distribution of spectrum similarity scores for *E. coli* between this analysis and Moritz *et al*. (**e** and **f**) Number of distinct peptides identified per protein identified by only, or at least one, prototypic peptide for (**e**) *H. bluephagenesis* TD01 and (**f**) *E. coli*.

The data in this study were processed through two pathways. Spectra from the MS^e^ data produced by the Xevo QtOF were extracted using proprietary programs apex3d and pep3d supplied by Waters. The latter of these output spectra to both a proprietary Waters format.bin file and a.mgf file that was converted to.mzML format to put through the trans-proteomic-pipeline workflow. The output of the trans-proteomics-pipeline fork was analysed using the MAYU^27^ tool to control the peptide-spectra match FDR at 2% and protein identification FDR at less than 5%. The 2% peptide-spectra FDR is a little higher than the 1% chosen for other recently published spectral libraries^34–37^, however as assay saturation curves (Fig. 2a) for both *E. coli* and *H. bluephagenesis* indicate the still stringent FDR threshold is closer to the assay saturation point. It is interesting to note the higher number of protein identifications achieved in the *E. coli* data over *H. bluephagenesis* in this plot. The more thorough annotation of the *E. coli* genome with 4391 gene products, the work of multiple groups over many iterations, vs *H. bluephagenesis* 3846 identified by automated analysis.

### Depth of proteome coverage

There were 3846 protein sequences in the *H. bluephagenesis*, and 4391 sequences in the *E. coli* fasta databases against which spectra were searched. The *E. coli* data serves as a quality control for the *H. bluephagenesis* data. A recently published comprehensive *E. coli* spectral library^32^ derived from whole cell lysate, over expressed proteins and synthetic peptides provides the highest quality reference point. That spectral library was re-mapped against the *E. coli* K12 proteome (uniprot UPID:UP000000625) used here. That spectral library covered 4090 of the 4391 entries in that proteome (93%). In comparison the spectral library presented here covers 1227 (30%) with 1135 (26%) quantified. The publicly available protein abundance database paxDB (https://pax-db.org) documents 1331 proteins that have been quantified by previous studies. Against this measure the present library covers 92% of these proteins and quantifies 85%. The depth of coverage against the paxDB database (Fig. 2b) shows higher abundance proteins are fully covered by the library and it is lower abundance proteins that are in varying proportions omitted. The coverage determined for the *E. coli* dataset with reference to community datasets assures that the *H. bluephagenesis* covers a similarly high proportion of the detectable and quantifiable region of the proteome. The is borne out by the similar coverage of the *H. bluephagenesis* proteome of the library by 1160 (30%) and quantification table 1063 (27%).

### Spectral library quality – iRT

Re-use of the spectral library for targeted data independent analysis requires retention time index and relative signal intensity within spectra to closely match newly acquired data. The recently published *E. coli* spectral library^32^ again provides a point of comparison. The library’s iRT annotation enables retention time comparison whilst library’s data acquisition on a Sciex Triple-TOF instrument enables comparison of spectra across instruments. The *E. coli* iRT in the quality control set closely match those in the Moritz library, with 90% of peptides attributed iRT differing by less than 4.4 and only 3% differing by more than 10. It is likely that 3% accounts for miss identification in either of the two libraries. Close agreement in iRT between the control and published *E. coli* libraries indicated the *H. bluephagenesis* is predictive of retention time on other instrument systems.

### Spectral library quality – spectra

The spectra for this library were acquired by MS^e^ on a Waters Xevo instrument. The wider applicability of these spectra to data acquired on other instruments, such as a Sciex Qtof, was assured by calculating a similarity score for all spectra between the Moritz land control library (Fig. 2d). The similarity score is based on the cosine of the angle between spectral and ranges between 0 and 1 with 1 being the highest quality match. By this score 50% of spectra have a similarity score greater than 0.75 and less than 25% have a similarity score lower than 0.5. The dissimilarity in lower scoring peptide spectra appears to be driven by the presence or absence of peaks rather than dramatic differences in the relative intensities of actually matched peaks. This may be due to the quite different signal processing steps required between DDA analysis in the Moritz library in which each spectrum is a snapshot of the fragmentation of an isolated peptide, and the MS^e^ technique used here in which picked peaks are algorithmically re-assembled into spectra by comparing elution profiles.

### Spectral library quality – DIALib-QC

The DIALib-QC tool^38^ provides calculates a set of 62 quality metrics for a spectral library describing it’s complexity, characteristic, coverage of modifications, completeness and correctness. The tool is implemented in the SWATHatlas website (http://www.swathatlas.org) and is a community standard. The DIALib-QC metrics for the openswath libraries for the Moritz library, control *E. coli* and *H. bluephagenesis* were generated (Supplementary Tables 1 and 2). The absolute numbers of ions, peptides and proteins covered by the libraries differ but the majority of metrics expressed in percentage terms are comparable across *H. bluephagenesis*, control *E. coli* and the Moritz library.

### Quantification quality

**Fig. 3:**
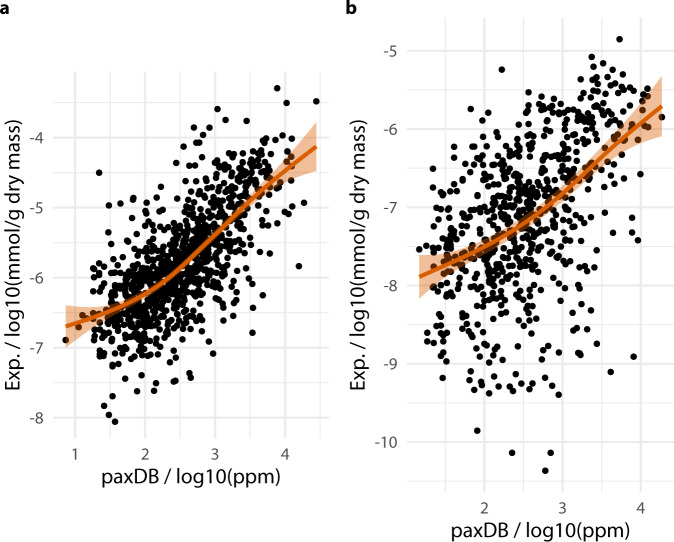
Comparison of protein quantification estimates against published data with generalised linear model overlayed (**a**) Control *E. coli* data plotted against PAXdb data. (**b**) *H. bluephagenesis* protein quantification plotted against quantification of most homologous *E. coli* protein as determined by Blast algorithm from paxDB data.

The quality of the quantification was assessed in two ways. First the control *E. coli* quantitative data were plotted against the PAXdb data (Fig. 3a). For the paxDB database quantifies 1331 proteins, the *E. coli* control dataset quantifies 1127 proteins with 939 proteins in common across the two datasets. The log-log plot shows that despite the inevitably dispersed data there is a clear trend across two orders of magnitude in estimated quantification. A further effort to confirm the quality of quantification in the *H. bluephagenesis* dataset was based on the principle that proteins with highly conserved sequences are likely to be critical to organism function and therefore likely to be maintained at similar cellular copy numbers in diverse species. Of the 1063 *H. bluephagenesis* proteins quantified, 690 were paired with an *E. coli* protein in the paxDB database via the blast algorithm as the top hitting match with a bitScore greater or equal to 50. The plot of *H. bluephagenesis* protein quantification against matched *E. coli* protein paxDB quantification (Fig. 3b) shows a similar correlation in quantification as *E. coli* vs *E. coli* with an expected wider dispersion given the differences between species. The agreement between these datasets suggest baseline expression data of the full set of 1050 quantified *H. bluephagenesis* protein is an accurate estimate of baseline expression.

### Coverage of biological function

The KO numbers of quantified *H. bluephagenesis* proteins (788 identifiers) were mapped against the KEGG database to indicate the coverage of biological function. This is particularly relevant to re-use of the data in support of adoption of the organism to bio manufacturing. The “Pathway” and “Module” tables are included as supplementary data (Supplementary Tables 3 and 4). Key observations from the map are that much of the core carbohydrate metabolism are covered, including all blocks from the citrate cycle and all but one module from the glycolysis pathway. There is substantial coverage of lipid, amino acid and nucleotide metabolism. Amongst cofactors there is good coverage of biosynthesis of thiamine, a coenzyme and riboflavin, a precursor to coenzymes flavin adenine dinucleotide and flavin mononucleotide. These coenzymes play a part in many metabolic pathways and ensuring their availability is critical to exploitation of such pathways for manufacturing.

## Supplementary information

DIA-QC Metrics for H bluephagenesis spectral library

Kegg analysis

Kegg analysis

Documented Halomonas Expressed Protein Index, Quantified Protein Profile and Spectral Library Producing Makefile

DIA-QC Metrics for E. coli spectral library

Table 1

## Data Availability

The script used to process raw data into the data is included as supplementary data, with the proteomics data deposition at pride^16^ and on the authors github page (https://github.com/M-Russell/HalomonasProcessingRepository).
