# Supplementary material for: Baseline proteomics characterisation of the emerging host biomanufacturing organism Halomonas bluephagenesis
Source: Sci Data. 2022 Aug 13;9:492. doi: 10.1038/s41597-022-01610-0 (PMC9376085; doi:10.1038/s41597-022-01610-0)
Supplement: Supplementary file 4 — Documented Halomonas Expressed Protein Index, Quantified Protein Profile and Spectral Library Producing Makefile [file 41597_2022_1610_MOESM4_ESM.pdf]

# Documented Halomonas Expressed Protein Index, Quantified Protein Profile and Spectral Library Producing Makefile

Matthew Russell

August, 2021

**Abstract**

Documentation of a makefile to reproduce protein index, protein quantification and spectral libraries for *Halomonas Bluephagenesis* TD01. A complete commentary on the accompanying makefile script to enable reproduction of libraries from raw mass spectrometry data files.

## Contents

**Introduction** **2**
- Introduction to Pipeline . . . 2
- Introduction to Make . . . 2

**System Setup** **3**
- PC Setup . . . 3
- Install All Required Programs . . . 3
- Executing Make . . . 4

**Documented Makefile for Pipeline** **4**
- Set Shell . . . 4
- Paths to Executables . . . 4
- Macros . . . 5
- Mass Spectrometry Raw Data Files . . . 6
- Intermediate Files . . . 8
- Fasta Files . . . 10
- Spesify Upload Packet . . . 11
- Spesify Targets . . . 11
- Recipies . . . 12
  - Generate Fasta Files . . . 12
  - Set Apex, Peptide3d and Mayu Variables . . . 14
    - Apex Settings . . . 14
    - Peptide3D Settings . . . 15
  - Peak Picking . . . 15
    - Apex Feature Picking . . . 15
    - Peptide3d Spectra Generation . . . 18
    - Merge . . . 19
  - IADB Search Whole Cell Lysate Merged Files . . . 19
  - TPP Pipeline for Spectral Library . . . 22
    - X!Tandem Search . . . 22
    - Interact and peptide prophet . . . 23
    - Mayu FDR Estimation . . . 24
    - Spectrast and Spectral Library Generation . . . 26

# Introduction

This document describes a data processing pipeline which generates data reported in paper "Baseline Proteomics Characterisation of Biomanufacturing Organism Halomonas Bluephagenesis". The pipeline is scripted in gnu-make and describes every process between raw data and the package submitted to PRIDE repository (https://doi.org/10.6019/PXD028156).

## Introduction to Pipeline

Data was acquired as $MS^e$ in which the instrument alternates between a low energy scan and a collision energy "ramp". The low energy scan captures precursor ions, the ramp captures product ion spectra. Data of this kind is not supported by opensource tools such as `openms` or `trans-proteomics pipeline` for spectra generation. So the Waters proprietary tools are used.

The proprietary tools that manage the pipeline as follows.

- Mass spectrometry data files are converted to feature lists by `apex3d.exe`.
- Precursor and product ions are matched by elution profile and combined into conventional spectra peak lists by `peptide3d64.exe`.
- Spectra are searched against a fasta database by `iadb.exe`.

It is possible to output additional information at each stage of the process. For example `apex3d.exe` is able to output a `.csv` file of all identified features and `peptide3d64.exe` can output a conventional `.mgf` file which may be submitted to other search engines. The final output of `iadb.exe` is a set of three `.csv` files summarising the outcome at a protein, peptide and fragment level.

After peakpicking data is searched and processed along two separate tracks. Protein expression is quantified using the "top three" method incorperated into Waters "iadb" search. Spectra obtained from the `peptide3d64.exe` are separately extracted as `.mgf` files and processed through the `X!Tandem` search engine and trans-proteomics-pathway to produce spectral libraries.

## Introduction to Make

The analysis pipeline is executed using `gun-make` also called `make`. The program `make` is well documented(https://www.gnu.org/software/make/manual). `Make` is principally used to compile software but is also useful to document bioinformatics pipelines. `Make` is a useful tool for scripting the "many-to-many" operations in which multiple mass spectrometry data files are each processed into a series of intermediate files followed by "many-to-one" operations where multiple intermediate files are combined into a summary output. `Make` facilitates writing commands for each operation in a single place for application to multiple files which ensures consistency and facilitates modification and experimentation with the pipeline as a whole.

The principle of `make` is that the program is supplied with a set of "rules" for converting "prerequisites" into "targets" by means of a "recipe". In this case the targets are a set of spectral libraries and the source files are MS datasets, `fasta` protein sequence libraries etc. Recipes take the general form:

```
target : prerequisite
  program -in prerequisite -out target
```

Recipes can be written in any order and `make` is able to chain them together to convert source files to target files via intermediate files. The following `make` file has been written with recipes in the sequence in which they would be run through the pipeline. When executed; make will parallelise the processing to speed up job completion.

`Make` is able to substitute text for macros which are defined `myMacro:=text` or `myMacroList:=element_01 element_02` and then called `${myMacro}` or `${myMacroList}`. It also has a set of built in functions that are

called in the general form: `$(function_name variable,param,param...)` which are able to manipulate variables, for example to change a file suffix, or change or remove a directory path. In this way it is possible to write rules convert raw data to intermediate files through an analysis pipeline without explicitly Term 1writing out all intermediate file names. Each recipe used for this project are briefly described and links to documentation supplied as encountered in the `make` file.

# System Setup

This section describes the programs that must be installed in order to run this pipeline.

## PC Setup

This pipeline was developed on a PC with spesification:

**Processor** Intel i7-7820X 3.6GHz 8 cores.

**RAM** 128 GB

**Operating System** Windows 10 2004 Build 19041.1165

Windows is required for Waters' proprietary tools. The `peptide3d` program and process consumes a lot of RAM, script detailed below prevents more than one instance of `peptide3d` running simultaneously to prevent the system running out of RAM. It is possible the pipeline will fail if run on a system with substantially less RAM than was used in development.

## Install All Required Programs

(1) If Waters executables (`apex3d`, `peptide3d`, `merge` and `iadb`) outlined above are not otherwise available download Progenisis QI for Proteomics and install, as this software installs the required executables. Here the executables from Waters' PLGS
(2) Download Rtools and install, this includes gnu tools `sed`, `awk`, `grep`, etc.
(3) If not present on your system download `gnu-make v4.3`. Compiled binaries can be found here: `https://github.com/mbuilov/gnumake-windows`. Earlier versions of gnu-make will not be compatible as the script uses features introduced in v4.3 such as grouped targets.
(4) If not present on your system download and install Trans-Proteomic Pipeline (TPP) this work was tested with v6.0.0.
(5) If not present on your system download and install x!tandem this work was tested with version ALANINE (2017.02.01). As an alternative the version of x!tandem distributed with the Trans-Proteomic Pipeline (TPP) may be used, however at present this is the older Jackhammer TPP (2013.06.15.1 - LabKey, Insilicos, ISB) version which was not used here.
(6) If not present on your system download and install seqkit this work was tested with v0.12.0.
(7) If not present on the system download and install openMS this work was tested with v2.4.0.
(8) A python distribution is required to run the msproteomicstools package. This work was tested with miniconda for python 3.7 with msproteomicstools installed as per the instructions given on the website. The minimal python installation enables just the requirements for this process to be installed with the versions required to replicate this work. Miniconda may be installed alongside other instances of python allowing this work to be repeated without disrupting a pre-existing system. During installation note the target directory and adjust if required. For single user installation this will look like: C:\Users\<user>\AppData\Local\Continuum\miniconda3, for all users it may be C:\ProgramData\Miniconda3. Note C:\ProgramData\ is a hidden directory on windows 10, navigate to it directly by entering path into windows explorer or set windows explorer to show hidden files.
(9) Install required python packages. To access the python command line, open a windows command window; navigate to the `Miniconda3\Scripts` directory; and type `activate` into the command line. Required packages will need to be installed from `pipy.org` through the python command line. The following commands entered into the anaconda prompt should install the required packages in the

versions used here. Alternatively the latest versions of these programs might be installed although that might require adjustments to processing commands below.

```
pip install numpy==1.15.3
pip install pymzml==0.7.5
pip install Biopython==1.72
pip install Cython==0.29.2 --install-option="--no-cython-compile"
pip install msproteomicstools==0.8.0
```

No further interaction with the python environment should be required.

### Executing Make

Gnu-make is executed from the command line. It it easiest to start a new `cmd` window in the project directory, set the `PATH` variable to just the location of gnu-make and then execute gnu-make on the script:

```
PATH="C:\Program Files\gmake"

gnumake-4.3-x64.exe -rR -j 8 -l 8 -f ./IntegratedAnalysis.mk  --output-sync all
```

The command line is documented on the gmake manual. Briefly: `-rR` turns off internal rules for compiling software; `-j` indicates the number of concurrent jobs permitted, select a lower number if limited processors or RAM available; `-l` checks load is below value specified before starting a new job, this provides some protection against overloading RAM which causes Waters programs to crash; `-f` indicates the file name of the makescript to run; `--output-sync` puts the output of each job on the console after the job finishes, if not set the output of concurrently running tasks will be ooutput concurrently and consequently unintelligible; `all` is the name of the "target" required by this build, alternatives are available for testing, see below.

## Documented Makefile for Pipeline

The following code blocks are combined into a single `gnu-make` script to run the pipeline.

### Set Shell

Each recipe in the make file is run in a "shell", that is a separate command line environment. On windows there is a choice of shell. This script used windows native executables from Waters to process the data and so the windows `cmd.exe` shell was chosen. It is likely that the script could be rewritten to run in the Windows Subsystem for Linux if required. However in this case all the `dos` commands such as `copy` and `ren` could need to be replaced with unix alternatives and paths changed to unix format.

```
SHELL=C:/Windows/System32/cmd.exe
```

Programs in the trans-proteomics-pipeline call other programs and particularly perl scripts. In order for these programs to be available they must be on the `PATH` for the shell when it is called by make. The `PATH` is set explicitly with the `export` to include the required trans-proteomics-pipeline directories.

The local for the shell is also explicitly set set to the default "C" local. Without setting this here `perl` may return a "locale" error which is harmless in itself but will stop the make script. Explicitly setting the locale to the default "C" locale by exporting `LC_ALL=C` to the shell prevents this error.

```
export PATH=C:\TPP\perl\bin;C:\TPP\bin
export LC_ALL=C
```

### Paths to Executables

Explicit control of software versions is assured by assigning the full path of each executable to a macro for each of the programs required for the pipeline. These must be edited to be correct for the system on which

the make script is to be run. Setting them explicity should also support reproducability since the version of software used here may be installed along side subsequent releases of the software buing used in future active research. Checking these are correct also acts as a check list to ensure system is set up correctly.

```
# Paths to the Waters executables
apex_path="C:\PLGS3.0.3\lib\apex3d\Apex3D64.exe"
peptide3d_path="C:\PLGS3.0.3\lib\apex3d\Peptide3D.exe"
IADB_path="C:\PLGS3.0.3\bin\iaDBs.exe"
mergeFractions_path="C:\PLGS3.0.3\bin\MergeFractions.exe"

# Paths to the sed and awk (gawk) used to process text files during library build
sed_path="C:\Rtools\bin\sed.exe"
awk_path="C:\Rtools\bin\gawk.exe"

# Path to 7 zip to zip data files for deposition.
zip7_path="C:\Program Files\7-Zip\7z.exe"

# Paths to spectrast etc
spectrast_path=\
"C:\Program Files\OpenMS-2.4.0\share\OpenMS\THIRDPARTY\SpectraST\spectrast.exe"
python_path="C:\ProgramData\Miniconda3\python.exe"
spectrast2tsv_path="C:\ProgramData\Miniconda3\Scripts\spectrast2tsv.py"
TargetedFileConverter_path="C:\Program Files\OpenMS-2.4.0\bin\TargetedFileConverter.exe"
perl_path="C:\TPP\perl\bin\perl.exe"
mayu_path="C:\TPP\bin\Mayu.pl"

# Paths to additional bioinformatics programs used in this analysis
DecoyDatabase_path="C:\Program Files\OpenMS-2.4.0\bin\DecoyDatabase.exe"
seqkit_path="C:\Program Files\seqKit_v0.12.0\seqkit.exe"


### TPP bits
Tandem2XML_path="C:\TPP\bin\Tandem2XML.exe"
xinteract_path="C:\TPP\bin\xinteract.exe"
PeptideProphet_path="C:\TPP\bin\PeptideProphetParser.exe"
perl_path="C:\TPP\perl\bin\perl.exe"
tandem_path="C:\Program Files\tandem-win-17-02-01-4\bin\tandem.exe"
msconvert_path="C:\Program Files\ProteoWizard\ProteoWizard 3.0.20002.36ad78f14\msconvert.exe"
Peptide3D2MGF_path="C:\PROGRA~1\Waters\Symphony\utils\PEPTID~1.EXE"


### Powershell
Powershell_path="C:\Windows\System32\WindowsPowerShell\v1.0\powershell.exe"
```

## Macros

In `make` macros are rules to generate a required instructions prior to execution. They may be used to generate output file names from an input file name, or vice versa. They may be used to generate entire recipe comands.

The following macro tests to see if required target directories exist and creates it if not. It is used at the start of most recipes to ensure the target directory is available.

```
define makeDir
if not exist $(subst /,\,$(abspath $(dir ${1}))) mkdir $(subst /,\,$(abspath $(dir ${1})))
```

```
endef
```

The following two macros turn relative file paths into absolute file paths, this allows relative paths to facilitate portability of the script but absolute paths to be provided to programs which require them. They also convert between unix-like forward slash "/" and windows like back slash "\" for those that need them.

```
define winify
$(subst /,\,$(abspath  ${1}))
endef

define unixify
$(subst \,/,$(abspath  ${1}))
endef
```

The following pair of macros between them find the position of a string within a gmake word list. The slightly esoteric formulation is a recursive macro _pos, which nibbles through a word list until it removes the matching element combined with pos which counts the remaining elements. The code enables the `pep3d` process to be run one file at a time as run concurrently multiple `pep3d` processes will consume all the RAM causing the processes to crash.

```
_pos = $(if $(findstring $1,$2),$(call _pos,$1,\
       $(wordlist 2,$(words $2),$2),x $3),$3)
pos = $(words $(call _pos,$1,$2))
```

The following macro calls powershell to obtain a UUID. This is used to pass a UUID to the IADB search.

```
UUID=$(shell ${Powershell_path} -Command "[guid]::NewGuid().ToString()")
```

## Mass Spectrometry Raw Data Files

The raw data acquired on the Xevo has been stored on a local hard drive.

Waters data are stored as multiple files inside a folder. `Make` can't treat a directory as a pre-requisite so each file is specified as the `_HEADER.TXT` file held in each of the directories and this element is removed when passed to the `apex3d` program.

The raw data file paths are concatenated into a single list of files so they can all be separately processed by a single command. This ensures consistency in data processing. Raw data directory paths will need to be adjusted to the location of downloaded data for re-use.

```
rawDirComps:=_CHRO001.DAT _CHRO002.DAT _CHRO003.DAT _CHRO004.DAT _CHRO005.DAT \
                            _CHROMS.INF _extern.inf _FUNC001.DAT _FUNC001.IDX _FUNC001.STS \
                            _FUNC002.DAT _FUNC002.IDX _FUNC002.STS _FUNC003.DAT _FUNC003.IDX \
                            _FUNC003.STS _FUNCTNS.INF _HEADER.TXT _INLET.INF

rawDirCompsNoDat:=_CHROMS.INF _extern.inf _FUNC001.IDX _FUNC001.STS \
                            _FUNC002.IDX _FUNC002.STS _FUNC003.IDX \
                            _FUNC003.STS _FUNCTNS.INF _HEADER.TXT _INLET.INF

rawDirEcoli_wcl=E:/massSpecData/xevo_FBRH_MR_Pro/Data/20_02_03_EcoliFractions/

rawFilesEcoli_wcl:= Ecoli_F01.raw Ecoli_F02_300ng.raw Ecoli_F03_300ng.raw \
                                Ecoli_F04_300ng.raw Ecoli_F05_300ng.raw \
                    Ecoli_F06_300ng.raw Ecoli_F07_300ng.raw \
                    Ecoli_F08_300ng.raw Ecoli_F09_300ng.raw \
                    Ecoli_F10_300ng.raw Ecoli_F11_300ng.raw \
```

```
                    Ecoli_F12_300ng.raw Ecoli_F13_300ng.raw \
                    Ecoli_F14_300ng.raw Ecoli_F15_300ng.raw \
                    Ecoli_F16_300ng.raw Ecoli_F17_300ng.raw \
                    Ecoli_F18_300ng.raw Ecoli_F19_300ng.raw \
                    Ecoli_F20_300ng.raw Ecoli_F21_300ng.raw \
                    Ecoli_F22_300ng.raw Ecoli_F25_300ng.raw \
                    Ecoli_F26_300ng.raw Ecoli_F27_300ng.raw \
                    Ecoli_F28_300ng.raw Ecoli_F29_300ng.raw \
                    Ecoli_F30_300ng.raw Ecoli_F31_300ng.raw

# Ecoli_F23_300ng.raw Ecoli_F24_300ng.raw

rawDirHalomonas_wcl=E:/massSpecData/xevo_FBRH_MR_Pro/Data/20_02_12_HaloFracs/


rawFilesHalo_wcl:= 05_Halo_F1.raw 06_Halo_F2.raw 07_Halo_F3.raw \
                                08_Halo_F4.raw 09_Halo_F5.raw 10_Halo_F6.raw \
                                11_Halo_F7.raw 12_Halo_F8.raw 13_Halo_F9.raw \
                                14_Halo_F10.raw 15_Halo_F11.raw 16_Halo_F12.raw \
                                17_Halo_F13.raw 18_Halo_F14.raw 19_Halo_F15.raw \
                                20_Halo_F16.raw 21_Halo_F17.raw 22_Halo_F18.raw \
                                23_Halo_F19.raw 25_Halo_F21.raw 26_Halo_F22.raw \
                                27_Halo_F23.raw 28_Halo_F24.raw 29_Halo_F25.raw \
                                30_Halo_F26.raw 31_Halo_F27.raw


rawFilesEcoliWCLPaths:=$(addsuffix /_HEADER.TXT, $(addprefix ${rawDirEcoli_wcl}, ${rawFilesEcoli_wcl}))
rawFilesHaloWCLPaths:=$(addsuffix /_HEADER.TXT, $(addprefix ${rawDirHalomonas_wcl}, ${rawFilesHalo_wcl}


rawFiles_All_wcl:=${rawFilesEcoliWCLPaths} ${rawFilesHaloWCLPaths}
```

```
############
# Edit for secretome

rawCellFracDir_1:=E:/massSpecData/xevo_FBRH_MR_Pro/Data/20_08_26_Hal_Ecoli_CellFracs/

rawCellFracDir_2:=E:/massSpecData/xevo_FBRH_MR_Pro/Data/21_02_19_MemPeri/

## Membrane

rawEcoli_Periplasm_Fracs_1:= mbpssmr9_01_EP_MSe_2uL.raw mbpssmr9_06_EP_MSe_2uL.raw mbpssmr9_11_EP_MSe_2

rawHalo_Periplasm_Fracs_1:= mbpssmr9_03_HP_MSe_2uL.raw mbpssmr9_08_HP_MSe_2uL.raw mbpssmr9_13_HP_MSe_2x



rawEcoli_Periplasm_Fracs_2:= mbpssmr9_EP.raw

rawFilesEcoliPeriPaths:=$(addsuffix /_HEADER.TXT, $(addprefix ${rawCellFracDir_1}, ${rawEcoli_Periplasm_
                                                 $(addsuffix /_HEADER.TXT, $(addprefix ${rawCellFracDir_

rawFilesHaloPeriPaths:=$(addsuffix /_HEADER.TXT, $(addprefix ${rawCellFracDir_1}, ${rawHalo_Periplasm_F
```

```
                                              $(addsuffix /_HEADER.TXT, $(addprefix ${rawCellFracDir_


rawFiles_All_peri:=${rawFilesEcoliPeriPaths} ${rawFilesHaloPeriPaths}
```

```
############
# Edit for secretome

rawSereteEcoliDir:=E:/massSpecData/xevo_FBRH_MR_Pro/Data/21_01_29_Secretome/

rawCellFracDir:=E:/massSpecData/xevo_FBRH_MR_Pro/Data/21_02_19_MemPeri/

rawEcoliSec:= mbpssmr9_ecoli_secretome_2uL_02.raw \
                            mbpssmr9_ecoli_secretome_5uL_01.raw \
                            mbpssmr9_ecoli_secretome_8uL_03.raw \
                            mbpssmr9_ecoli_secretome_highConc_5uL_01.raw

rawHaloSec:= 21_02_19_SecretomeHalo_10uL.raw 21_02_19_02_SecretomeHalo.raw


rawFilesEcoliSecPaths:=$(addsuffix /_HEADER.TXT, $(addprefix ${rawSereteEcoliDir}, ${rawEcoliSec}))
rawFilesHaloSecPaths:=$(addsuffix /_HEADER.TXT, $(addprefix ${rawCellFracDir}, ${rawHaloSec}))


rawFiles_All_sec:=${rawFilesEcoliSecPaths} ${rawFilesHaloSecPaths}
```

With all the raw data specified they are grouped for convenience into lists of all files, and of each bacteria species separatly.

```
rawFiles_All:=${rawFilesEcoliSecPaths} ${rawFilesHaloSecPaths} ${rawFilesEcoliPeriPaths} ${rawFilesHalo


rawFiles_Ecoli:=${rawFilesEcoliSecPaths} ${rawFilesEcoliPeriPaths} ${rawFilesEcoliWCLPaths}

rawFiles_Halo:=${rawFilesHaloSecPaths} ${rawFilesHaloPeriPaths} ${rawFilesHaloWCLPaths}

rawDir:=${rawFilesEcoli_wcl} ${rawFilesHalo_wcl} ${rawEcoli_Periplasm_Fracs_1} ${rawHalo_Periplasm_Fracs
```

## Intermediate Files

Lists of intermediate files are useful for setting put the rules for converting intermediate files. Here the names of the required intermediate files are determined from the raw data file names. `Apex3d` output files are put into sub dir `apex`, `peptide3d` to sub dir `pep3d` and `iadb` files to subdir `iadb`. The useful outputs of `iadb`, the tables of protein, peptide and fragment level summary data, are output to sub dir `iadb\tables` although that is handled in the recipes.

```
apexDir:=./apex/
pep3dDir:=./pep3d/
pep3dMergedDir:=./pep3d/merge/
iadbDir:=./iadb/
iadbTabDir:=./iadb/tables/
TPP:=./tpp_mayu/
```

```
# Apex paths:
apexPaths:=$(addprefix ${apexDir},$(notdir $(rawFiles_All:.raw/_HEADER.TXT=_Apex3D.bin)))
# $(info first apex $(firstword ${apexPaths}))

# Apex paths WCL:
apexPaths_wcl:= $(addprefix ${apexDir},$(notdir $(rawFiles_All_wcl:.raw/_HEADER.TXT=_Apex3D.bin)))

# Apex paths periplasm:
apexPaths_peri:=$(addprefix ${apexDir},$(notdir $(rawFiles_All_peri:.raw/_HEADER.TXT=_Apex3D.bin)))

# Apex paths periplasm:
apexPaths_sec:=$(addprefix ${apexDir},$(notdir $(rawFiles_All_sec:.raw/_HEADER.TXT=_Apex3D.bin)))

# Pep3d Paths:
pep3dPaths:=$(addprefix ${pep3dDir},$(notdir $(rawFiles_All:.raw/_HEADER.TXT=_Pep3D_Spectrum.bin)))
pep3dAll:=${pep3dPaths} ${pep3dMergedFiles}

# Merged pep3dFiles
pep3dMergedFiles := ./pep3d/merged_halo_wcl_Pep3D_Spectrum.bin \
                                        ./pep3d/merged_ecoli_wcl_Pep3D_Spectrum.bin

#IADB paths
## Merged files:
iadb_mg_bin:=$(abspath $(addprefix ${iadbDir},$(notdir $(pep3dMergedFiles:_Pep3D_Spectrum.bin=_IA_workfl
iadb_mg_tabFrag:=$(abspath $(addprefix ${iadbTabDir},$(notdir $(pep3dMergedFiles:_Pep3D_Spectrum.bin=_I
iadb_mg_tabPep:=$(abspath $(addprefix ${iadbTabDir},$(notdir $(pep3dMergedFiles:_Pep3D_Spectrum.bin=_IA
iadb_mg_tabProt:=$(abspath $(addprefix ${iadbTabDir},$(notdir $(pep3dMergedFiles:_Pep3D_Spectrum.bin=_I

## All IADB files:
iadb_all:=$(sort ${iadb_mg_bin} ${iadb_mg_tabFrag} ${iadb_mg_tabPep} ${iadb_mg_tabProt})


# mzXML
pep3dEcoilPaths_mzXML:=$(addprefix ./pep3d/,$(notdir $(rawFiles_Ecoli:.raw/_HEADER.TXT=_Pep3D_Spectrum.
pep3dHaloPaths_mzXML:=$(addprefix ./pep3d/,$(notdir $(rawFiles_Halo:.raw/_HEADER.TXT=_Pep3D_Spectrum.mz
all_mzXML:=${pep3dEcoilPaths_mzXML} ${pep3dHaloPaths_mzXML}

# x!tandem
all_tandem:= $(all_mzXML:.mzXML=.t.xml)

# pepXML
pep3dEcoliPaths_pepXML:=$(addprefix ./pep3d/,$(notdir $(rawFiles_Ecoli:.raw/_HEADER.TXT=_Pep3D_Spectrum
pep3dHaloPaths_pepXML:=$(addprefix ./pep3d/,$(notdir $(rawFiles_Halo:.raw/_HEADER.TXT=_Pep3D_Spectrum.p
all_pepXML:=${pep3dEcoliPaths_pepXML} ${pep3dHaloPaths_pepXML}

# mzid
all_mzid:=$(all_mzXML:.mzXML=.t.xml.mzid)

# interact
all_interact:=./pep3d/interact_ecoli_all.pep.xml ./pep3d/interact_halo_all.pep.xml

# prophet
all_prophet:=./pep3d/interact_all_ecoli_prophet.pep.xml ./pep3d/interact_all_halo_prophet.pep.xml
```

```
# mayuFiles
mayuOut:= ./tpp_mayu/mayuOut_all_halo_psm_protFDR0.05_t_1.07.csv \
                    ./tpp_mayu/mayuOut_all_ecoli_psm_protFDR0.05_t_1.07.csv \
                    ./tpp_mayu/mayuOut_all_halo_main_1.07.csv \
                    ./tpp_mayu/mayuOut_all_ecoli_main_1.07.csv \
                    ./tpp_mayu/mayuOut_all_ecoli_plot_psm_protFDR0.04_t_1.07.csv    \
                    ./tpp_mayu/mayuOut_all_ecoli_plot_main_1.07.csv \
                    ./tpp_mayu/mayuOut_all_halo_plot_psm_protFDR0.04_t_1.07.csv \
                    ./tpp_mayu/mayuOut_all_halo_plot_main_1.07.csv
```

The spectral libraries output in various formats defined here:

```
nonConsLib:=./tpp_mayu/ecoli_all.splib ./tpp_mayu/halo_all.splib

ConsLib:=./tpp_mayu/ecoli_all_cons.splib ./tpp_mayu/halo_all_cons.splib ./tpp_mayu/ecoli_SWATHatlas2020

TsvLib_pk:=$(ConsLib:.splib=_pk.tsv)

TsvLib_os:=$(ConsLib:.splib=_os.tsv)

targetSplib:= ${nonConsLib} ${ConsLib} ${TsvLib_pk} ${TsvLib_os}

uploadSplib:= ${nonConsLib} $(nonConsLib:.splib=.sptxt) ${TsvLib_pk} ${TsvLib_os}
```

### Fasta Files

The `.fasta` protein database files are specified here.

Also specified are a pair of `.fasta` databases of common contaminants to draw-off likely contaminating spectra and a file containing a the peptides set as retention-time and quantitative standards concatenated into a single pseudo-protein.

```
# organism fasta files
fasta_ecoli=D:/fasta/Ecoli_MG1655_UP000000625.fasta
fasta_halo=./ini/halAndy/Halomonas_Hal1chrom.feature_protein.fasta

# iRT and contaminant fasta files
fasta_iRT=./ini/irtfusion_quant.fasta
fasta_iRT_single=./ini/irtfusionSingleProt.fasta
fasta_cRAP=./ini/crap.fasta
fasta_MPI=./ini/contaminants.fasta
fasta_NoDumCont=./ini/NoDup_cont.fasta

# derived fasta files used in the analysis
fastaForSearchEcoli=./ini/fastaForSearchEcoli.fasta
fastaForSearchHalo=./ini/fastaForSearchHalo.fasta
fastaForSearchEcoli_decoy=./ini/fastaForSearchEcoli_decoy.fasta
fastaForSearchHalo_decoy=./ini/fastaForSearchHalo_decoy.fasta
fastaForSpectrastEcoli=./ini/fastaForSpectrastEcoli.fasta
fastaForSpectrastHalo=./ini/fastaForSpectrastHalo.fasta
fastaForSearchHalo_xtand=./ini/fastaForSearchHalo_xtand.fasta
fastaForSearchEcoli_xtand=./ini/fastaForSearchEcoli_xtand.fasta
fastaForSearchHalo_xtand_decoy=./ini/fastaForSearchHalo_xtand_decoy.fasta
```

```
fastaForSearchEcoli_xtand_decoy=./ini/fastaForSearchEcoli_xtand_decoy.fasta

fastaForTandem=${fastaForSearchHalo_xtand_decoy} ${fastaForSearchEcoli_xtand_decoy}


RTindex=./ini/iRTPeptides_huge.txt

# List of fasta files to be included in the data submission
uploadFasta:= ${fastaForTandem} ${fastaForSearchEcoli} ${fastaForSearchHalo} ${fasta_halo} ${fasta_ecol
```

The specified `.fasta` files are combined so that:

- The two contaminant databases are combined and given a common `cont_` prefix to protein names.
- The contaminant and iRT databases are combined with the strain specific database for halomonas C3001.
- All annotation apart from accession number is stripped from the sequence annotation.

The `.fasta` files are also converted to a table format for easy import and use in R.

## Spesify Upload Packet

This section specifies the files which are to be uploaded to Pride and copies them to a specific folder for upload.

```
uploadDir:=D:/HaloLibForUpload/


#uploadRaw_zip:=$(addsuffix /_HEADER.TXT,$(addprefix ${uploadDir},$(notdir $(rawFiles_All:.raw/_HEADER.

#uploadRaw:=$(foreach raw,${rawDir},$(addprefix ${uploadDir},$(foreach,comp,${rawDirComps},$(addsuffix

thisMakeFile:=$(lastword $(MAKEFILE_LIST))

#uploadRawDir:=$(foreach raw,${rawDir},$(addprefix ${uploadDir},${raw}))
uploadRawDir:=$(foreach raw,${rawDir},$(addprefix ${uploadDir},$(raw:.raw=.raw.zip)))
uploadRaw:=$(foreach comp,${rawDirCompsNoDat},$(addsuffix /${comp},${uploadRawDir}))
# $(info allRawComp: ${uploadRaw})

intermediateFilesForUpload:= ${uploadFasta} ${uploadSplib} ${all_pepXML} ${all_interact} ${all_prophet}
uploadFileList:=$(addprefix ${uploadDir},$(notdir ${intermediateFilesForUpload}))

upload_mzid:= $(addprefix ${uploadDir},$(notdir $(all_mzid:_Pep3D_Spectrum.t.xml.mzid=.mzid)))

uploadTargets := ${uploadFileList} ${upload_mzid} ${uploadRaw}
```

## Spesify Targets

A critical part of a `makefile` is specification of the targets. Providing recipes exists to build intermediate and final target files from input files all these targets will be produced. The targets specified in `all` will be built. This is assured by making the special variable `.PHONY` dependent on `all` because all pre-requisites of `.PHONY` must always be rebuilt. The target `.PHONY` is the first target defined in the file and an invocation of make on the makefile will built that target if no other target is given on the comand line. Other targets may be spesified on the gnu-make invoking comand line, the targets `buildSet` for example `buildApex` are provided for trouble shooting to enable each step of the process to be built in turn. The special target `.ONESHELL` is also set here to specify that all lines in a recipe be run in the same shell invocation rather than each in a

separate invocation. Running in a single shell is important for a few recipes where variables are set in one line and used in subsequent lines in the recipe.

```
allTargets:= $(sort ${apexPaths}) ${pep3dPaths} ${iadb_all} ${targetSplib} ${mayuOut}
allUpload:= ${uploadTargets} ${rawFiles_All_zip}

.PHONY: all \
buildMZID buildInteract buildProphet buildMayu buildSplib buildUpload \
buildTandem buildApex buildPep3d  testBuildApex testBuildPep3d testBuild_mzXML \
testBuildTandem buildIadb buildPep3dMerge buildUploadRaw buildUploadInt

all: ${mayuOut} ${allTargets} ${allUpload} ${spectrastTargets}


$(info all targets: $(sort ${allTargets} ${allUpload}))

.ONESHELL:

buildFasta : ${uploadFasta}
buildApex : ${apexPaths}
buildPep3d : ${pep3dPaths}
buildPep3dMerge : ${pep3dMergedFiles}
buildIadb : ${iadb_all}
buildTandem : ${all_tandem}
buildMZID : ${all_mzid}
buildInteract : ${all_interact}
buildProphet : ${all_prophet}
buildMayu : ${mayuOut}
buildSplib : ${targetSplib}
buildUpload : ${uploadTargets}
buildUploadRaw : ${uploadRaw}
buildUploadInt : ${uploadFileList}

apexTestFile:=$(lastword ${apexPaths})
pep3dTestFile:=$(lastword ${pep3dPaths})
mzXMLTestFile:= ./pep3d/31_Halo_F27_Pep3D_Spectrum.mzXML
tandemTestFile:= ./pep3d/31_Halo_F27_Pep3D_Spectrum.t.xml


testBuildApex:${apexTestFile}
testBuildPep3d:${pep3dTestFile}
testBuild_mzXML:${mzXMLTestFile}
testBuildTandem:${tandemTestFile}
```

## Recipies

### Generate Fasta Files

```

decoy_string=reverse_
cont_string=cont_

$(fasta_ecoli:.fasta=_clean.fasta) : ${fasta_ecoli}
    ${sed_path} -e 's/[[:blank:]].*//g' $< > $@
```

```
$(fasta_cRAP:.fasta=_clean.fasta) : ${fasta_cRAP}
    ${sed_path} -e 's/^>/^>${cont_string}/g' $< > $@


$(fasta_MPI:.fasta=_clean.fasta) : ${fasta_MPI}
    ${sed_path} -e 's/^>/^>${cont_string}/g;s/[[:blank:]].*//g' $< > $@

${fasta_NoDumCont} : $(fasta_MPI:.fasta=_clean.fasta) $(fasta_cRAP:.fasta=_clean.fasta)
    type $(call winify, $^) > $(call winify, .\ini\tmp_$(notdir $@))
    ${seqkit_path} rmdup --by-seq --dup-num-file ./ini/duplicatedCont.txt -o $(call winify, .\ini\tmp_$
    del $(call winify, .\ini\tmp_$(notdir $@))


${fastaForSearchEcoli} : $(fasta_ecoli:.fasta=_clean.fasta) ${fasta_NoDumCont} ${fasta_iRT}
    type $(call winify, $^) > $(call winify, $@)

${fastaForSearchHalo} : ${fasta_halo} ${fasta_NoDumCont} ${fasta_iRT}
    type $(call winify, $^) > $(call winify, $@)
    ${sed_path} -i 's/[[:blank:]].*//g' $@


${fastaForSearchEcoli_xtand} : $(fasta_ecoli:.fasta=_clean.fasta) ${fasta_NoDumCont} ${fasta_iRT_single
    type $(call winify, $^) > $(call winify, $@)

${fastaForSearchHalo_xtand} : ${fasta_halo} ${fasta_NoDumCont} ${fasta_iRT_single}
    type $(call winify, $^) > $(call winify, $@)
    ${sed_path} -i 's/[[:blank:]].*//g' $@

${fastaForSpectrastEcoli} : $(fasta_ecoli:.fasta=_clean.fasta) ${fasta_iRT_single}
    type $(call winify, $^) > $(call winify, $@)

${fastaForSpectrastHalo} : ${fasta_halo} ${fasta_iRT_single}
    type $(call winify, $^) > $(call winify, $@)
    ${sed_path} -i 's/[[:blank:]].*//g' $@



./ini/HaloFasta.tsv: ${fastaForSearchHalo}
    ${seqkit_path} fx2tab $< > $@

./ini/EcoliFasta.tsv: ${fastaForSearchEcoli}
    ${seqkit_path} fx2tab $< > $@


${fastaForSearchHalo_decoy} : ${fastaForSearchHalo}
    ${DecoyDatabase_path} -in $^ -out $@ -decoy_string ${decoy_string}


${fastaForSearchEcoli_decoy} : ${fastaForSearchEcoli}
    ${DecoyDatabase_path} -in $^ -out $@ -decoy_string ${decoy_string}
```

```
${fastaForSearchHalo_xtand_decoy} : ${fastaForSearchHalo_xtand}
    ${DecoyDatabase_path} -in $^ -out $@ -decoy_string ${decoy_string}


${fastaForSearchEcoli_xtand_decoy} : ${fastaForSearchEcoli_xtand}
    ${DecoyDatabase_path} -in $^ -out $@ -decoy_string ${decoy_string}
```

### Set Apex, Peptide3d and Mayu Variables

It is possible to use `makefile` variables to set the values of switches on executables. For full executables for Apex and Peptide3d see Apex Feature Picking and Peptide3d Spectra Generation. However during pipeline refinement the following parameters to which the process is quite sensitive were taken out to this section for ease of adjustment.

**Apex Settings** The Apex3d program has many settings which are set below:

```
# Whole cell lysate APEX settings

bCSVOutput_wcl:=1
apex_stRT_wcl:=12
apex_endRT_wcl:=80
le3DThreshCounts_wcl:=10
he3DThreshCounts_wcl:=25
chromFWHMMin_wcl:=0.45
apex_binThresh_wcl:=650
apexTrackSNRThreshold_wcl:=2.25
chromFilterScaleFactor_wcl:=0.7
driftFilterScaleFactor_wcl:=0.7
msFilterScaleFactor_wcl:=0.7
apexTrackFilterWide_wcl:=0
apexTrackSNRMeasFWHM_wcl:=1
minScansPerPeak_wcl:=7
msResolution_wcl:=20000
noFilteredIons_wcl:=1
bEnableTempCal_wcl:=1
```

```
# Periplasm apex settings

bCSVOutput_peri:=1
apex_stRT_peri:=12
apex_endRT_peri:=80
le3DThreshCounts_peri:=10
he3DThreshCounts_peri:=25
chromFWHMMin_peri:=0.4
apex_binThresh_peri:=550
apexTrackSNRThreshold_peri:=2.0
chromFilterScaleFactor_peri:=0.7
driftFilterScaleFactor_peri:=0.7
msFilterScaleFactor_peri:=0.7
apexTrackFilterWide_peri:=0
apexTrackSNRMeasFWHM_peri:=2.5
minScansPerPeak_peri:=6
msResolution_peri:=20000
```

```
noFilteredIons_peri:=1
bEnableTempCal_peri:=1
```

```
# Apex - from HaloSpecLib

bCSVOutput_sec:=1
apex_stRT_sec:=12
apex_endRT_sec:=80
le3DThreshCounts_sec:=10
he3DThreshCounts_sec:=25
chromFWHMMin_sec:=0.4
apex_binThresh_sec:=550
apexTrackSNRThreshold_sec:=2
chromFilterScaleFactor_sec:=0.7
driftFilterScaleFactor_sec:=0.7
msFilterScaleFactor_sec:=0.7
apexTrackFilterWide_sec:=0
apexTrackSNRMeasFWHM_sec:=1
minScansPerPeak_sec:=6
msResolution_sec:=20000
noFilteredIons_sec:=1
bEnableTempCal_sec:=1
```

**Peptide3D Settings** As with Apex3d Peptide3d has a many settings. A few of these were adjusted during pipeline development and are set here:

```
minHEIntenThreshold:=10
minLEIntenThreshold:=25
msResolution:=20000
disableSingleHE:=0
```

### Peak Picking

The Apex3d and peptide3d algorithms between them work to pick peaks from the raw data, de-isotope them, relate precursor and product ions and produce spectra peaklists for database searching. I am not entirely clear how the tasks are split. I think Apex does the initial peak picking and feature detection and peptide3d associates precursor and product ions and determines peptide mass from multiply charged precursors and outputs a de-isotope peak list. I need to find some documentation to properly understand this.

**Apex Feature Picking** The Apex algorithm is run with settings closely mirroring those run by PLGS. The major difference is substantial effort put into optimising the low-energy and high-energy thresholds that appear critical in getting high numbers of peptides hits.

For whole cell lysate:

```

define rawApex_rule
${1} : ${2}
    $(call makeDir,${1})
    ${apex_path} -pRawDirName $(call winify,${2}) \
                    -outputDirName $(dir $(call winify,${1})) \
                    -outputUserDirName $(dir $(call winify,${1}))user \
                    -bEnableLockMass 1 \
```

```
                    -noFilteredIons ${noFilteredIons} \
                    -bCSVOutput ${bCSVOutput_wcl} \
                    -lockmassZ1 556.2771 \
                    -lockMassToleranceAMU 0.25 \
                    -le3DThresholdCounts ${le3DThreshCounts_wcl} \
                    -he3DThresholdCounts ${he3DThreshCounts_wcl} \
                    -startingRTMin ${apex_stRT_wcl} \
                    -endingRTMin ${apex_endRT_wcl} \
                    -chromFWHMMin ${chromFWHMMin_wcl} \
                    -binIntenThreshold ${apex_binThresh_wcl} \
                    -WriteXML 0 \
                    -isADC 1 \
                    -bEnableCuda 0 \
                    -adcCalc2.128 1 \
                    -apexTrackSNRThreshold ${apexTrackSNRThreshold_wcl} \
                    -apexTrackMaxIterations 10 \
                    -chromFilterScaleFactor ${chromFilterScaleFactor_wcl} \
                    -driftFilterScaleFactor ${driftFilterScaleFactor_wcl} \
                    -msFilterScaleFactor ${msFilterScaleFactor_wcl} \
                    -apexTrackFilterWide ${apexTrackFilterWide_wcl} \
                    -apexTrack3DCombine 0 \
                    -4DReportFilterIntensity 1 \
                    -apexTrackSNRMeasFWHM ${apexTrackSNRMeasFWHM_wcl} \
                    -startingMassAmu 265 \
                    -endingMassAmu 2000 \
                    -msResolution ${msResolution_wcl} \
                    -minScansPerPeak ${minScansPerPeak_wcl} \
                    -bEnableTempCal ${bEnableTempCal_wcl}

endef


$(foreach rawFile, ${rawFiles_All_wcl},$(eval $(call rawApex_rule,\
$(addprefix ${apexDir},$(notdir $(rawFile:.raw/_HEADER.TXT=_Apex3D.bin))),\
$(rawFile:.raw/_HEADER.TXT=.raw))))
```

For periplasm:

```
define rawApex_rule
${1} : ${2}
    $(call makeDir,${1})
    ${apex_path} -pRawDirName $(call winify,${2}) \
                    -outputDirName $(dir $(call winify,${1})) \
                    -outputUserDirName $(dir $(call winify,${1}))user \
                    -bEnableLockMass 1 \
                    -noFilteredIons ${noFilteredIons} \
                    -bCSVOutput ${bCSVOutput_peri} \
                    -lockmassZ1 556.2771 \
                    -lockMassToleranceAMU 0.25 \
                    -le3DThresholdCounts ${le3DThreshCounts_peri} \
                    -he3DThresholdCounts ${he3DThreshCounts_peri} \
                    -startingRTMin ${apex_stRT_peri} \
                    -endingRTMin ${apex_endRT_peri} \
                    -chromFWHMMin ${chromFWHMMin_peri} \
```

```
                    -binIntenThreshold ${apex_binThresh_peri} \
                    -WriteXML 0 \
                    -isADC 1 \
                    -bEnableCuda 0 \
                    -adcCalc2.128 1 \
                    -apexTrackSNRThreshold ${apexTrackSNRThreshold_peri} \
                    -apexTrackMaxIterations 10 \
                    -chromFilterScaleFactor ${chromFilterScaleFactor_peri} \
                    -driftFilterScaleFactor ${driftFilterScaleFactor_peri} \
                    -msFilterScaleFactor ${msFilterScaleFactor_peri} \
                    -apexTrackFilterWide ${apexTrackFilterWide_peri} \
                    -apexTrack3DCombine 0 \
                    -4DReportFilterIntensity 1 \
                    -apexTrackSNRMeasFWHM ${apexTrackSNRMeasFWHM_peri} \
                    -startingMassAmu 265 \
                    -endingMassAmu 2000 \
                    -msResolution ${msResolution_peri} \
                    -minScansPerPeak ${minScansPerPeak_peri} \
                    -bEnableTempCal ${bEnableTempCal_peri}

endef


$(foreach rawFile, ${rawFiles_All_peri},$(eval $(call rawApex_rule,\
$(addprefix ${apexDir},$(notdir $(rawFile:.raw/_HEADER.TXT=_Apex3D.bin))),\
$(rawFile:.raw/_HEADER.TXT=.raw))))
```

For secretome:

```

define rawApex_rule
${1} : ${2}
    $(call makeDir,${1})
    ${apex_path} -pRawDirName $(call winify,${2}) \
                    -outputDirName $(dir $(call winify,${1})) \
                    -outputUserDirName $(dir $(call winify,${1}))user \
                    -bEnableLockMass 1 \
                    -noFilteredIons ${noFilteredIons} \
                    -bCSVOutput ${bCSVOutput_sec} \
                    -lockmassZ1 556.2771 \
                    -lockMassToleranceAMU 0.25 \
                    -le3DThresholdCounts ${le3DThreshCounts_sec} \
                    -he3DThresholdCounts ${he3DThreshCounts_sec} \
                    -startingRTMin ${apex_stRT_sec} \
                    -endingRTMin ${apex_endRT_sec} \
                    -chromFWHMMin ${chromFWHMMin_sec} \
                    -binIntenThreshold ${apex_binThresh_sec} \
                    -WriteXML 0 \
                    -isADC 1 \
                    -bEnableCuda 0 \
                    -adcCalc2.128 1 \
                    -apexTrackSNRThreshold ${apexTrackSNRThreshold_sec} \
                    -apexTrackMaxIterations 10 \
                    -chromFilterScaleFactor ${chromFilterScaleFactor_sec} \
```

```
                    -driftFilterScaleFactor ${driftFilterScaleFactor_sec} \
                    -msFilterScaleFactor ${msFilterScaleFactor_sec} \
                    -apexTrackFilterWide ${apexTrackFilterWide_sec} \
                    -apexTrack3DCombine 0 \
                    -4DReportFilterIntensity 1 \
                    -apexTrackSNRMeasFWHM ${apexTrackSNRMeasFWHM_sec} \
                    -startingMassAmu 265 \
                    -endingMassAmu 2000 \
                    -msResolution ${msResolution_sec} \
                    -minScansPerPeak ${minScansPerPeak_sec} \
                    -bEnableTempCal ${bEnableTempCal_sec}

endef

$(foreach rawFile, ${rawFiles_All_sec},$(eval $(call rawApex_rule,\
$(addprefix ${apexDir},$(notdir $(rawFile:.raw/_HEADER.TXT=_Apex3D.bin))),\
$(rawFile:.raw/_HEADER.TXT=.raw))))
```

**Peptide3d Spectra Generation** The peptide3d algorithm is run with as close as possible to the PLGS defaults. Some changes have been made. There was an issue with peptide3d "hanging" with some files. The process would consume all the RAM and never complete. Documentation on this from Waters suggested altering some settings as noted in the code below.

The recipe uses the pipe | as part of its dependency instruction. All dependencies after this symbol are "order-only" dependencies. This means they will not force re-running of a recipe if they themselves are updated, however if they are to be updated in this run they must be rebuild prior to the current target. The feature is used here to ensure each pep3d file in the complete list of pep3d files has all subsequent pep3d files in the list as order only dependencies. The result is that only a single pep3d file may be produced at a time to prevent this particular operation overwhelming available RAM and crashing the process.

```

define pep3d_rule
${1} $(1:_Pep3D_Spectrum.bin=_Pep3DAMRT.mgf) &: ${2} | ${3} ${4}
    $(call makeDir,${1})
    ${peptide3d_path} -inputFilename $(call winify,${2}) \
                                        -outputDirName $(call winify,$(dir ${1})) \
                                        -WriteAllIonsToCSV 0 \
                                        -outputUserDirName $(call winify,$(dir ${1}))\user \
                                        -WriteXML 0 \
                                        -minLEMHPlus 350 \
                                        -minHEMHPlus 265 \
                                        -minHEIntenThreshold ${minHEIntenThreshold} \
                                        -minLEIntenThreshold ${minLEIntenThreshold} \
                                        -msResolution ${msResolution} \
                                        -clusMzFwhmFraction 0.1428 \
                                        -amrtMzFwhmFraction 0.219 \
                                        -amrtChFWHMFraction 0.25 \
                                        -disableSingleHE ${disableSingleHE} \
                                        -bMGFOutput

endef

$(foreach apexFile, ${apexPaths},\
$(eval $(call pep3d_rule,\
```

```
$(subst apex,pep3d,$(apexFile:_Apex3D.bin=_Pep3D_Spectrum.bin)),\
${apexFile},\
$(filter-out $(subst apex,pep3d,$(apexFile:_Apex3D.bin=_Pep3D_Spectrum.bin)),\
$(wordlist $(call pos,\
$(subst apex,pep3d,$(apexFile:_Apex3D.bin=_Pep3D_Spectrum.bin)),\
$(subst apex,pep3d,$(apexPaths:_Apex3D.bin=_Pep3D_Spectrum.bin))),\
$(words ${apexPaths}),\
$(subst apex,pep3d,$(apexPaths:_Apex3D.bin=_Pep3D_Spectrum.bin)))),\
$(filter-out $(subst apex,pep3d,$(apexFile:_Apex3D.bin=_Pep3DAMRT.mgf)),\
$(wordlist $(call pos,\
$(subst apex,pep3d,$(apexFile:_Apex3D.bin=_Pep3DAMRT.mgf)),\
$(subst apex,pep3d,$(apexPaths:_Apex3D.bin=_Pep3DAMRT.mgf))),\
$(words ${apexPaths}),\
$(subst apex,pep3d,$(apexPaths:_Apex3D.bin=_Pep3DAMRT.mgf))))\
)))
```

**Merge** The fractionated whole cell lysate peak lists produced by peptide3d are merged prior to search and quantification by IADB.

```
define pep3dFileFromRaw
    $(addprefix ./pep3d/,$(notdir $(1:.raw/_HEADER.TXT=_Pep3D_Spectrum.bin)))
endef

merge_halo_wcl.ini : $(call pep3dFileFromRaw,${rawFilesHaloWCLPaths})
    $(file >$@) $(foreach bin,$^,$(file >>$@,$(call winify,${bin})))

merge_ecoli_wcl.ini : $(call pep3dFileFromRaw,${rawFilesEcoliWCLPaths})
    $(file >$@) $(foreach bin,$^,$(file >>$@,$(call winify,${bin})))


./pep3d/merged_halo_wcl_Pep3D_Spectrum.bin : merge_halo_wcl.ini
    ${mergeFractions_path} -inputFileSpectrums $(call winify,$<) \
                                               -outputUserDirName ".\pep3d\merge_halo_wcl" \
                                               -WriteXML 0 \
                                               -outputFile $(call winify, $@)

./pep3d/merged_ecoli_wcl_Pep3D_Spectrum.bin : merge_ecoli_wcl.ini
    ${mergeFractions_path} -inputFileSpectrums $< \
                                               -outputUserDirName ".\pep3d\merge_ecoli_wcl" \
                                               -WriteXML 0 \
                                               -outputFile $(call winify, $@)
```

### IADB Search Whole Cell Lysate Merged Files

The search is run through the Waters search system `iadb.exe`. The program requires an input of spectra from a Waters `.bin` file; a `.fasta` database to search; and a `.xml` file specifying the search parameters. The latter was exported from the PLGS gui and edited below.

```
define searchSets
<?xml version="1.0" encoding="UTF-8"?>
<WORKFLOW_TEMPLATE TITLE="FBRH_BacteriaSearch" UUID="${UUID}" WORKFLOW_TEMPLATE_ID="_15731280752780_529[illegible]
    <PROTEINLYNX_QUERY TYPE="Databank-search">
        <DATABANK_SEARCH_QUERY_PARAMETERS>
            <SEARCH_ENGINE_TYPE VALUE="PLGS"/>
```

```
            <SEARCH_TYPE NAME="Electrospray-Shotgun"/>
            <IA_PARAMS>
                <FASTA_FORMAT VALUE="Long Description"/>
                <PRECURSOR_MHP_WINDOW_PPM VALUE="20"/>
                <PRODUCT_MHP_WINDOW_PPM VALUE="30"/>
                <NUM_BY_MATCH_FOR_PEPTIDE_MINIMUM VALUE="7"/>
                <NUM_PEPTIDE_FOR_PROTEIN_MINIMUM VALUE="1"/>
                <NUM_BY_MATCH_FOR_PROTEIN_MINIMUM VALUE="12"/>
                <PROTEIN_MASS_MAXIMUM_AMU VALUE="300000"/>
                <FALSE_POSITIVE_RATE VALUE="1"/>
                <AQ_PROTEIN_ACCESSION VALUE=""/>
                <AQ_PROTEIN_MOLES VALUE="-1"/>
                <MANUAL_RESPONSE_FACTOR VALUE="-1"/>
                <DIGESTS>
                    <ANALYSIS_DIGESTOR MISSED_CLEAVAGES="1">
                        <AMINO_ACID_SEQUENCE_DIGESTOR NAME="Trypsin" UUID="138fcbbc-4399-4fdb-ae63-9310
                            <CLEAVES_AT AMINO_ACID="K" POSITION="C-TERM">
                                <EXCLUDES AMINO_ACID="P" POSITION="N-TERM"/>
                            </CLEAVES_AT>
                            <CLEAVES_AT AMINO_ACID="R" POSITION="C-TERM">
                                <EXCLUDES AMINO_ACID="P" POSITION="N-TERM"/>
                            </CLEAVES_AT>
                        </AMINO_ACID_SEQUENCE_DIGESTOR>
                    </ANALYSIS_DIGESTOR>
                </DIGESTS>
                <MODIFICATIONS>
                    <ANALYSIS_MODIFIER STATUS="FIXED">
                        <MODIFIER MCAT_REAGENT="No" NAME="Carbamidomethyl+C">
                            <MODIFIES APPLIES_TO="C" DELTA_MASS="57.0215" TYPE="SIDECHAIN"/>
                        </MODIFIER>
                    </ANALYSIS_MODIFIER>
                    <ANALYSIS_MODIFIER ENRICHED="FALSE" STATUS="VARIABLE">
                        <MODIFIER MCAT_REAGENT="No" NAME="Oxidation+M">
                            <MODIFIES APPLIES_TO="M" DELTA_MASS="15.9949" TYPE="SIDECHAIN"/>
                        </MODIFIER>
                    </ANALYSIS_MODIFIER>
                </MODIFICATIONS>
            </IA_PARAMS>
        </DATABANK_SEARCH_QUERY_PARAMETERS>
    </PROTEINLYNX_QUERY>
</WORKFLOW_TEMPLATE>
endef

params.xml : #.FORCE
    $(file > params.xml,${searchSets})
```

The search execution is set up below. A rule is created to search every given `pep3d` file and output a matching search result.

```
define IadbFromPep3d
$(abspath $(addprefix ${iadbDir},$(notdir $(1:_Pep3D_Spectrum.bin=_IA_workflow.bin))))
endef

define pepCSVfromPep3d
```

```
$(abspath $(addprefix ${iadbTabDir},$(notdir $(1:_Pep3D_Spectrum.bin=_IA_final_peptide.csv))))
endef

define protCSVfromPep3d
$(abspath $(addprefix ${iadbTabDir},$(notdir $(1:_Pep3D_Spectrum.bin=_IA_final_protein.csv))))
endef

define fragCSVfromPep3d
$(abspath $(addprefix ${iadbTabDir},$(notdir $(1:_Pep3D_Spectrum.bin=_IA_final_fragment.csv))))
endef


define search_rule
$(call IadbFromPep3d,${1}) $(call fragCSVfromPep3d,${1}) $(call pepCSVfromPep3d,${1}) $(call protCSVfrom[illegible]
    $(call makeDir,$(call IadbFromPep3d,${1}))
    $(call makeDir,$(call fragCSVfromPep3d,${1}))
    ${IADB_path} -paraXMLFileName params.xml \
                                -pep3DFileName $(call winify,${1}) \
                                -proteinFASTAFileName $(call winify,${2}) \
                                -proteinForAbsQuan "iRT_Quant" \
                                -protAmountForAbsQuanInFMol 434 \
                                -WriteXML 0 \
                                -allowHeMultiZ 1 \
                                -bAutoCalibrate 1 \
                                -outputDistractionProteins 1 \
                                -fragmentTypes BY \
                                -bEnablePPMCalc 1 \
                                -protFalsePositiveRateMax 100 \
                                -outPutDirName $(call winify,$(dir $(call IadbFromPep3d,${1}))) \
                                -outputUserDirName $(call winify,$(dir $(call fragCSVfromPep3d,${1})))
endef





$(eval $(call search_rule,\
./pep3d/merged_ecoli_wcl_Pep3D_Spectrum.bin,$\
${fastaForSearchEcoli_decoy}))


$(eval $(call search_rule,\
./pep3d/merged_halo_wcl_Pep3D_Spectrum.bin,$\
${fastaForSearchHalo_decoy}))


$(eval $(call search_rule,\
./pep3d/merged_ecoli_all_Pep3D_Spectrum.bin,$\
${fastaForSearchEcoli_decoy}))


$(eval $(call search_rule,\
./pep3d/merged_halo_all_Pep3D_Spectrum.bin,$\
```

```
${fastaForSearchHalo_decoy}))
```

### TPP Pipeline for Spectral Library

The trans-proteomic pipeline is used to process `.mgf` files produced by peptide3d above into a spectral library. The results of the TPP are cross referenced with the iadb search to yield a high quality list of quantified proteins.

**X!Tandem Search** The first step is to convert `.mgf` files into mzXML files which can be searched by X!tandem:

```
define mzXML_Rule
${1} : ${2}
    $(call makeDir,${1})
    ${msconvert_path} ${2} --mzXML -o $(dir ${1}) --outfile $(notdir ${1})
endef

$(foreach mzXMLfile, ${all_mzXML},\
        $(eval \
            $(call mzXML_Rule,$\
                ${mzXMLfile},$\
                $(mzXMLfile:_Pep3D_Spectrum.mzXML=_Pep3DAMRT.mgf)\
        )\
))
```

Each of the resulting `.mzXML` files is then searched with x!tandem as below. The process is a little complex. For each mzxml file a copy is made of the standard xtandem paramater file. This copy is then edited with required spectrum file, target file name and fasta search file. After the search is complete, which outputs a tandem xml and an mzid file, the paramater file is deleted.

```
define Xtandem_Rule
${1}  $(1:.t.xml=.t.xml.mzid) &: ${2} ${3} .\ini\default_input.xml .\ini\Xtandem_params.xml ${fastaForTa
    $(call makeDir,${1})
    copy $(call winify, .\ini\Xtandem_params.xml) $(call winify, $(1:.t.xml=_params.xml))
    ${sed_path} -i "s|spectrumFile|$(subst \,\\,${2})|"  $(1:.t.xml=_params.xml)
    ${sed_path} -i "s|outFile|$(subst \,\\,${1})|" $(1:.t.xml=_params.xml)
    ${sed_path} -i "s|taxonomyForSearch|$(subst \,\\,${3})|" $(1:.t.xml=_params.xml)
    ${tandem_path} $(1:.t.xml=_params.xml)
    del /F $(call winify, $(1:.t.xml=_params.xml))
    if exist sed* del sed*
endef



$(foreach specFile, ${pep3dEcoilPaths_mzXML},\
        $(eval \
            $(call Xtandem_Rule,$\
                $(specFile:.mzXML=.t.xml),${specFile},$\
                ./ini/taxonomy_ecoli.xml)\
        )\
)
```

```
$(foreach specFile, ${pep3dHaloPaths_mzXML},\
        $(eval \
            $(call Xtandem_Rule,$\
                $(specFile:.mzXML=.t.xml),${specFile},$\
                ./ini/taxonomy_halo.xml)\
        )\
)
```

The `.t.xml` files are then converted into `.pepXML` files to be processed by the rest of the TPP:

```
define Xtandem_pep_xml_Rule
${1}: ${2}
    $(call makeDir,${1})
    ${Tandem2XML_path} $(call winify,${2}) $(call winify,${1})
endef

$(foreach pepXML, ${pep3dEcoliPaths_pepXML},\
    $(eval \
        $(call Xtandem_pep_xml_Rule,\
                ${pepXML},\
                $(pepXML:.pep.xml=.t.xml)\
            )\
    )\
)

$(foreach pepXML, ${pep3dHaloPaths_pepXML},\
    $(eval \
        $(call Xtandem_pep_xml_Rule,\
                ${pepXML},\
                $(pepXML:.pep.xml=.t.xml)\
            )\
    )\
)
```

**Interact and peptide prophet** The `pepXML` files are then combined into single pepXML files using TPP `xinteract`:

```
define xinteract_Rule
${1} : ${2}
    $(call makeDir,${1})
    if exist $(call winify,${1}), del /F $(call winify,${1})
    ${xinteract_path} -eT \
                            -MONO \
                            -E${3} \
                            -l7 \
                            -nP \
                            -D${3} \
                            -d${decoy_string} \
                            -N${1} ${2}
endef


$(eval \
```

```
            $(call xinteract_Rule,$\
                    ./pep3d/interact_halo_all.pep.xml,$\
                    ${pep3dHaloPaths_pepXML},$\
                    ${fastaForSearchHalo_xtand_decoy},$\
                ))\
)


$(eval \
            $(call xinteract_Rule,$\
                    ./pep3d/interact_ecoli_all.pep.xml,$\
                    ${pep3dEcoliPaths_pepXML},$\
                     ${fastaForSearchEcoli_xtand_decoy},$\
                ))\
)
```

The scores for the peptides are then adjusted using peptide prophet:

```
define peptideProphet_Rule
${1} : ${2}
    $(call makeDir,${1})
    if exist $(2:.pep.xml=_tmp.pep.xml) del /F $(2:.pep.xml=_tmp.pep.xml)
    if exist $(call winify,${1}), del /F $(call winify,${1})
    copy $(call winify,${2}) $(call winify,$(2:.pep.xml=_tmp.pep.xml))
    if exist ${1} del /F ${1}
    ${PeptideProphet_path} $(call winify,$(2:.pep.xml=_tmp.pep.xml)) \
    ACCMASS PPM MINPROB=0.05 DECOY=${decoy_string} \
    DECOYPROBS NONPARAM NONTT IGNORECHG=1 IGNORECHG=4 \
    IGNORECHG=5 IGNORECHG=6 IGNORECHG=7
    ren $(call winify,$(2:.pep.xml=_tmp.pep.xml)) $(notdir ${1})
endef

$(eval \
            $(call peptideProphet_Rule,$\
                    ./pep3d/interact_all_ecoli_prophet.pep.xml,$\
                    ./pep3d/interact_ecoli_all.pep.xml)\
)


$(eval \
            $(call peptideProphet_Rule,$\
                    ./pep3d/interact_all_halo_prophet.pep.xml,$\
                    ./pep3d/interact_halo_all.pep.xml)\
)
```

**Mayu FDR Estimation** Mayu is a tool that estimates peptide and protein false discovery rates. Mayu is actually run twice here. One time with settings defined in `mayu_Rule` to find the correct score to set for spectra import into spectrast. The second time with settings defined in `mayu_Rule_plot` making calculations to higher FDR to produce data for figures presented in the paper.

```

define mayu_Rule
${1} ${2} &: ${3} ${4}
    $(call makeDir,${1})
```

```
    ${perl_path} ${mayu_path} \
    -A $(call winify,${3}) \
    -M $(subst _psm_protFDR0.05_t_1.07.csv,,$(call winify,${1})) \
    -C $(call winify,${4}) \
    -E ${decoy_string} \
    -G 0.02 \
    -H 49 \
    -I 1 \
    -P protFDR=0.05:t
endef



$(eval \
            $(call mayu_Rule,\
                ./tpp_mayu/mayuOut_all_halo_psm_protFDR0.05_t_1.07.csv,\
                ./tpp_mayu/mayuOut_all_halo_main_1.07.csv,\
                ./pep3d/interact_all_halo_prophet.pep.xml,\
                ${fastaForSearchHalo_xtand_decoy})\
)



$(eval \
            $(call mayu_Rule,\
                ./tpp_mayu/mayuOut_all_ecoli_psm_protFDR0.05_t_1.07.csv,\
                ./tpp_mayu/mayuOut_all_ecoli_main_1.07.csv,\
                ./pep3d/interact_all_ecoli_prophet.pep.xml,\
                ${fastaForSearchEcoli_xtand_decoy})\
)



define mayu_Rule_plot
${1} ${2} &: ${3} ${4}
    $(call makeDir,${1})
    ${perl_path} ${mayu_path} \
    -A $(call winify,${3}) \
    -M $(subst _psm_protFDR0.04_t_1.07.csv,,$(call winify,${1})) \
    -C $(call winify,${4}) \
    -E ${decoy_string} \
    -H 100 \
    -PmFDR \
    -I 1 \
    -G 0.05 \
    -P protFDR=0.04:t
endef

$(eval \
            $(call mayu_Rule_plot,\
                ./tpp_mayu/mayuOut_all_halo_plot_psm_protFDR0.04_t_1.07.csv,\
                ./tpp_mayu/mayuOut_all_halo_plot_main_1.07.csv,\
                ./pep3d/interact_all_halo_prophet.pep.xml,\
```

```
                ${fastaForSearchHalo_xtand_decoy})\
)



$(eval \
            $(call mayu_Rule_plot,\
                ./tpp_mayu/mayuOut_all_ecoli_plot_psm_protFDR0.04_t_1.07.csv,\
                ./tpp_mayu/mayuOut_all_ecoli_plot_main_1.07.csv,\
                ./pep3d/interact_all_ecoli_prophet.pep.xml,\
                ${fastaForSearchEcoli_xtand_decoy})\
)
```

**Spectrast and Spectral Library Generation** The integrated search results are now converted into a spectral library with spectrast. This takes `pepXML` files generated above, filters them to control FDR against the mayu result files and applies an iRT index.

```
define spectrast_Rule
${1} $(1:.splib=.sptxt) &: ${2} ${3} ${4}
    if not exist $(dir ${1}) mkdir $(dir ${1})
    $$(eval mayuCutoff := $$(shell ${awk_path} -F, "BEGIN{min=1}{if($$$$5<=min)  min=$$$$5}END{print mi[illegible]
    $$(file > $$(dir ${4})mayuCutoff.txt,$${mayuCutoff})
    ${spectrast_path} -V \
                -cN$(call unixify,$(basename ${1})) \
                -cICID-QTOF \
                -c_IRR \
                -c_IRT$(call unixify,${3}) \
                -cP$${mayuCutoff} \
                -co \
                -c_RDY"${decoy_string}" \
                $(call unixify,${2})
endef

# WCL

$(eval $(call spectrast_Rule,\
    ./tpp_mayu/halo_all.splib,\
    ./pep3d/interact_all_halo_prophet.pep.xml,${RTindex},\
    ./tpp_mayu/mayuOut_all_halo_psm_protFDR0.05_t_1.07.csv\
))

$(eval $(call spectrast_Rule,\
    ./tpp_mayu/ecoli_all.splib,\
    ./pep3d/interact_all_ecoli_prophet.pep.xml,${RTindex},\
    ./tpp_mayu/mayuOut_all_ecoli_psm_protFDR0.05_t_1.07.csv\
))




#################
#################
#################
```

```
define spectrastCons_Rule
${1} $(1:.splib=.sptxt) &: ${2} ${3}
    if not exist $(dir ${1}) mkdir $(dir ${1})
    ${spectrast_path} -V \
                -cN$(basename ${1}) \
                -cICID-QTOF \
                -c_ANN \
                -cf"iRT<190" \
                -cu \
                -c_NPK7 \
                -cD${3} \
                -cAC \
                ${2}
endef



$(eval $(call spectrastCons_Rule,./tpp_mayu/ecoli_all_cons.splib,./tpp_mayu/ecoli_all.splib,${fastaForSp
$(eval $(call spectrastCons_Rule,./tpp_mayu/ecoli_SWATHatlas2020.splib,./litData/EcoliSWATHatlas2020/Ec
$(eval $(call spectrastCons_Rule,./tpp_mayu/halo_all_cons.splib,./tpp_mayu/halo_all.splib,${fastaForSpe


#################
#################
#################
```

The spectral libraries produced by spectrast are now converted into `.tsv` format for ease of use with other DIA tools such as openSWATH and peakview.

```
define spectrast2tsv_Rule
${1} : ${2}
    if not exist $(dir ${1}) mkdir $(dir ${1})
    ${python_path} ${spectrast2tsv_path}  \
                        -l 100,2000 \
                        -s b,y \
                        -x 1,2 \
                        -g -17.03,-18.0 \
                        -o 3 \
                        -n 20 \
                        -p 0.05 \
                        -d -e \
                        -k openswath \
                        -a ${1} \
                        ${2}
endef

$(foreach splib2conv, ${ConsLib},\
        $(eval \
                $(call spectrast2tsv_Rule,\
                        $(basename ${splib2conv})_os.tsv,\
                        $(basename ${splib2conv}).sptxt,\
                    )\
            )\
```

```
)
```

```
define spectrast2tsv_Rule
${1} : ${2}
    if not exist $(dir ${1}) mkdir $(dir ${1})
    ${python_path} ${spectrast2tsv_path}  \
                        -l 100,2000 \
                        -s b,y \
                        -x 1,2 \
                        -g -17.03,-18.0 \
                        -o 3 \
                        -n 20 \
                        -p 0.05 \
                        -d -e \
                        -k peakview \
                        -a ${1} \
                        ${2}
endef

$(foreach splib2conv, ${ConsLib},\
        $(eval \
                $(call spectrast2tsv_Rule,\
                        $(basename ${splib2conv})_pk.tsv,\
                        $(basename ${splib2conv}).sptxt,\
                    )\
            )\
)
```

## Create Upload Package

Copy raw data from the various data directories to the upload

```

define zip7raw
$(foreach comp,${rawDirCompsNoDat},$(addsuffix /${comp},${uploadDir}$(notdir $(1:.raw/_HEADER.TXT=.raw.[illegible]
    ${zip7_path} u $(call winify,$(dir $(firstword $(foreach comp,${rawDirComps},$(addsuffix /${comp},$[illegible]
    if exist $(call winify,$(dir $(firstword $(foreach comp,${rawDirComps},$(addsuffix /${comp},${uploa[illegible]
endef

rawFileTest:=$(firstword ${rawFiles_All})


$(info testUpload: $(rawFileTest:.raw/_HEADER.TXT=.raw))

#$(foreach raw, ${rawFiles_All},$(info $(call zip7raw,${raw})))

$(foreach raw, ${rawFiles_All},\
        $(eval \
                $(call zip7raw,\
                        ${raw} \
                    )\
            )\
)
```

```
define copyToUpload_MZID
${1} : ${2}
    copy /y $(subst /,\,$(abspath  ${2})) $(subst /,\,$(abspath  ${1}))
endef

# # $(info MZID: ${all_mzid})

$(foreach file, ${all_mzid},\
        $(eval \
                $(call copyToUpload_MZID,\
                        $(addprefix ${uploadDir},$(notdir $(file:_Pep3D_Spectrum.t.xml.mzid=.mzid))),\
                        ${file}\
                    )\
            )\
)
```

```

define copyToUpload
$(addprefix ${uploadDir},$(notdir ${1})) : ${1}
    copy /y $(call winify, ${1}) $(call winify,${uploadDir})
endef


$(foreach file, ${intermediateFilesForUpload},\
        $(eval \
                $(call copyToUpload,\
                        ${file}\
                    )\
            )\
)
```
